# Permittivity extraction of sandy soils through uncalibrated and thickness-independent meniscus-free measurements using a hollow coaxial line

**DOI:** 10.1038/s41598-025-32731-2

**Published:** 2026-01-22

**Authors:** Ugur C. Hasar, Huseyin Korkmaz, Yunus Kaya

**Affiliations:** 1 Department of Electrical and Electronics Engineering, Gaziantep University, Gaziantep, 27310 Turkey; 2 Applied Electromagnetics Research Laboratory, Gaziantep University, Gaziantep, 27310 Turkey; 3 Department of Electronics and Automation, Bayburt University, Bayburt, 69010 Turkey

**Keywords:** Engineering, Materials science, Physics

## Abstract

Measurement techniques for accurate moisture level determination of various soil conditions are becoming popular in recent studies. Microwave-driven techniques, as positioned into indirect methods, could be tailored for precise and real-time detection of soil moisture. However, improper implementation of calibration techniques or usage of imperfect calibration standards required for precise measurements by these techniques presents a challenging limitation for their widespread usage. Besides, meniscus formation on top of soil samples with a sufficient moisture level could adversely influence the performance of these techniques. In this study, we present a microwave extraction method for eliminating the requirement of a formal calibration procedure and additionally removing the effect of meniscus on measurements through relative measurements of sandy soil samples while maintaining accurate permittivity extraction with no information of sample thickness. To achieve our goal, a mathematical framework unifying wave-cascading matrix and state-transition matrix presentations is proposed for permittivity determination from three measurement configurations (empty line, the (sandy) soil sample sandwiched between two identical plugs, and a shifted version of this plug-sample-plug configuration) and their inverses implemented by port switching operation. The proposed method is first validated by permittivity measurements of two liquid samples (distilled water and methanol). Then, permittivity measurements of two sandy soil samples (having more than 90% sand content) were conducted using Type-N-to-EIA 1-5/8” coaxial lines (with no universal calibration standard) over 0.05 GHz–3.0 GHz. From these measurements, it is noted that our meniscus-free method determines permittivity with a maximum absolute difference of 3.4% (evaluated from five independent measurements) while another similar method, suffering from meniscus presence, extracts permittivity with a maximum absolute difference of 4.0%. This indicates a clear advantage of our method for measurements of sandy soil samples having meniscus formation. We then utilized to construct a calibration curve, compared with other calibration curves using by other methods, allowing prediction of moisture volume content ($\theta _v$) of the tested sandy soil samples from measured permittivity data whose conformity analysis implemented by the three-pole Debye model and the Mironov-Fomin model. It is observed that this calibration curve could be utilized to predict $\theta _v$ within $5\%$ margin from measured permittivity data.

## Introduction

Moisture content (water) in soil is an important parameter directly affecting the plant growth and soil erosion and thus considered as one of the most influential parameters used to develop strategies for improving agricultural productivity^1–4^. In addition, soil water is a key factor governing mass and energy exchange at air-soil interfaces^5^. Furthermore, soil moisture is a key role in the studies related to water balance, slope stability, and performance of geotechincal structures such as pavements and foundations^6^. Measuring techniques for moisture (water) content in soils can be categorized into direct methods and indirect methods^7–10^. The thermo-gravimetric method, as one of the most widely utilized direct methods in the literature, is a moisture level measuring tool, based on the difference between weights of the sample in air-dry state and the same sample with some moisture^3^. This technique, however, is time-consuming, not applicable for real-time measurements, destructive (tested soil cannot be reused for subsequent tests due to deterioration in the structure), and not feasible for continues dynamic evaluation of soil moisture level. In addition, it requires intensive labor and laboratory environment to perform measurements. On the other hand, indirect methods are proposed to eliminate some of the aforementioned limitations of the thermo-gravimetric method. These methods rely on associating a measured non-moisture parameter (e.g., heat dissipation, scattering, resistance, dielectric constant) with the moisture content within soil samples. There are a variety of indirect methods, each having unique advantages and some limitations, available in the literature. The tensiometer technique is a primary technique for relating a matric (capillary tension) potential, defined as the portion of water potential attributable to the attraction of the soil matrix, to soil moisture content^11^. Its working principle is based on negative tension created in the tensiometer moisture unit after this unit is buried into the soil. Although it is inexpensive, applicable for in situ or lab, and easy-to-install, and has no heath risk, it has small spatial variability and is limited to low-moisture soils (less situated for fine textures soils) due to pressure scale (0–1 atm). Besides, as another indirect method, the neutron probe method determines soil moisture by detecting scattered neutrons generated from a neutron source due to collision with various atomic ions in the soil. Even though is is applicable for in situ applications, has a fast response (smaller response time), and can perform continuous soil moisture measurements at fixed points withing mining or destroying soil texture pattern without any hysteresis, it has a potential health risk (limiting its widespread usage) and is mainly feasible for subsurface soil because its accuracy is highly restricted to soil samples with small spatial variations. Moreover, resistive sensors can be utilized to evaluate soil moisture level by quantifying soil resistance measured between electrodes in a soil since a higher soil moisture accompanies with lower soil resistivity. It is relatively inexpensive and applicable for in situ or laboratory measurements, and does not contain any health risk. Nonetheless, it requires a calibration curve established between soil moisture content and measured associated electrical resistivity (time-consuming) and has some failure results for saline soils.

Falling into indirect soil moisture measuring tool category based on dielectric measurements, the time domain reflectometry (TDR)^12–15^, the ground-penetrating radar (GPR)^16^, and microwave sensing methods^17–28^ can also be utilized for real-time applications without involvement of much labor^5^. TDR measurements are relevant to propagation velocity change of generated pulses due to a change in soil moisture in the soil. Even if they have fast response, could be adapted for non-invasive analysis, and do not pose any health problem, they necessitate the usage for custom-designed probes and thus are influenced by probe-oriented problems limiting its application to non-highly saline soils^29^. What is more, it is frequency-dependent due to finite rise time of the generated pulses. In a similar manner, the GPR methodology exploits the change in reflection and transmission properties of a wide-frequency band signal (e.g., 1 MHz–1 GHz) due to a change in high-frequency impedance in the soil sample. It is chiefly susceptible to moisture content changes and has the advantage of relating these changes to empirical formula (e.g., Topp’s equation^12^). Nevertheless, the accuracy of GPR measurements can degrade with uncontrolled environmental conditions including heterogeneity present in the soil sample, leading to a significant decrease in the accuracy for highly conductive soil samples^20^. Additionally, they are feasible for subsurface sensing. Microwave sensing methods, as indirect methods alternative for TDR and GPR measurements, are attractive in the sense that they provide accurate measurement results for various soil samples while maintaining real-time measurements with minimal invasion^30–33^. A brief comparison of direct and indirect methods used in evaluation of soil moisture is presented in Table 1. A comprehensive review about various measurement techniques of soil moisture and dielectric models for moisture determination of soils can be found in recent studies^7–10,34,35^.

**Table 1:** A brief comparison of direct and indirect methods used in evaluation of soil moisture.

Methodology	Application	Working	Reaction	Advantages	Limitations
	Area	Mechanism	Time		
Thermo-gravimetric sensors	Laboratory	Evaporation	$\approx$ 24 h	Accurate, standard, and no-risk to health	Time-consuming, destructive, and non-feasible for continuous measurements
Tensiometer sensors	In situ or lab	Tension or potential	2-3 h	Inexpensive, easy-to-install, no-risk to health	Small spatial variability and chiefly applicable for sandy soils
Neutron probe sensors	In situ	Neutron scattering	1-2 min	Smaller response time, feasible for continuous measurements at fixed points, and no hysteresis	Health risk and implementable for subsurface soil analysis
Resistive sensors	In situ or lab	Electrical resistance	2-3 h	Inexpensive and no health risk	Calibration curve requirement (time-consuming) and failure in saline soils
TDR sensors	In situ or lab	Dielectric constant	$\approx$ 1 min	No health risk, adapted to non-invasive measurements, and fast response	A custom-designed probe, failure in highly saline soils, and frequency-dependence
GPR sensors	In situ or lab	Dielectric constant	Depend on calibration	No health risk, highly sensitive, and relatively simple calibration curve based on well-established formula	Accuracy degrade in uncontrolled environmental conditions, applicable for subsurface sensing, and failure in highly saline soils
Microwave sensors	In situ or lab	Dielectric constant and attenuation constant	Depend on calibration	No health risk, accurate, and real-time measurements	Calibration curve requirement and measurement system calibration

There are diverse microwave methods available in the literature, which could be categorized into resonant methods and non-resonant methods^36^. Between these categories, our concern in this study is non-resonant microwave methods which provide broadband material characterization. Planar microwave sensors^28^, open-ended coaxial or waveguide measuring systems^17,37,38^, free-space methods^39,40^, and coaxial-line or waveguide measurements^18–27,41–54^ can be considered as examples for non-resonant microwave methods. Among these methods, coaxial-line or waveguide methods give flexibility of measuring electromagnetic properties of wide range of materials including heterogeneous materials. Nonetheless, they require implementation of an involved calibration process, which otherwise results in a decrease in measurement accuracy, leading to an improvement of measurement consistency. Provided that appropriate calibration standards are available, calibration of microwave vector network analyzer (VNA) setups including probes is not a major challenge. A calibration procedure, as an example, based on five different coaxial line standards was implemented in the calibration process in a recent study^19,20^. However, the accuracy of soil moisture evaluation using coaxial line measurements is utterly dependent on the proper application of calibration standards which are generally expensive and may result in residual signals interfering with electromagnetic response of the sample. In addition, for some measurement environments, calibration standards are not standardized. For example, the measurement setups involving coaxial lines with Type-N-to-EIA 1-5/8” adapters^19,55^ do not have standardized calibration standards to implement a proper calibration procedure. Therefore, calibration-independent microwave methods^55–71^ could be not only implemented to mitigate the effects of imperfect calibration standards on measurements but also be applied for some measurement environments with no specific standardized calibration standards.

The calibration-independent methods in the studies^56,57,60–62,64,65^ are applicable for electromagnetic characterization of solid materials only. Besides,the calibration-independent methods in the studies^55,58,59,63,66–71^ are applicable for material characterization of granular/liquid materials, which are then our focus in this study, because a soil sample with a certain moisture level will have combination of granular composition and liquid state. When the calibration-independent methods in the studies^55,58,59,63,66–71^ are examined, it is noted that only those in the studies^63,67,68,71^ try to eliminate the meniscus formation on the surface of samples. Meniscus formation could reduce the accuracy of material characterization if its effect is not considered in the theoretical model or its effect is not eliminated for circumstances whose theoretical model is not present^54^. The method in the study^71^ tackles the problem of meniscus problem in semi-open test fixtures by utilizing only one plug to fix the position of the liquid or keep the liquid sample. Although effective, this method requires correct positioning of the test fixture in vertical position (no tilt) and assumes that three liquid samples with different amounts have the same meniscus state. Additionally, different from the calibration-dependent method in the study^50^, this calibration-independent method necessitates correct amount of sample increment (and thus the sample thickness) in the electromagnetic characterization process.

The calibration-independent methods in the studies^63,67,68^ could be equally utilized to measure electromagnetic properties of liquid samples sandwiched by two plugs, thereby eliminating the meniscus effects. Such an exercise can eliminate the problem arising from non-perfect vertical positioning of semi-infinite test fixtures (tilt issue) as well as removing the need for the same meniscus state, provided that the effects of the air bubbles between the plug and the sample are minimized. Nonetheless, the calibration-independent methods in the studies^63,67,68,71^ require information of sample thickness before the calibration procedure. In this research, we propose another non-resonant microwave method for electromagnetic parameter extraction of sandy soil samples from uncalibrated scattering (S-) parameter measurements without resorting the sample thickness while eliminating the effect of meniscus formation by means of using two plugs. It will be shown in our study that the effect of air bubbles, which could be considered as the potential limitation, between the plug and the sample could be eliminated providing that the sandy soil sample is poured slowly and uniformly. Besides, when the calibration-independent techniques in the literature are considered^58,59,63,66–71^, it is observed that either the transfer matrix (TM) (ABCD matrix) presentation or the wave-cascading matrix (WCM) presentation is utilized in the theoretical model. Different from these theoretical models, in our present study, as our second contribution, we integrate the state-transition matrix (STM) presentation into the WCM presentation to have more strong mathematical model for the analysis of eliminating the meniscus formation.

The organization of the remainder of the manuscript is as follows. First, the new formalism based on WCM and STM presentations is presented in “The method”. Next, the results of numerical analyses for examining the solution space are given in “Numerical and accuracy analyses”. Then, validation of the proposed method by permittivity measurements of two liquid samples (distilled water and methanol) is discussed in “Measurement setup and validation”. After, permittivity results of two soil samples with different moisture levels determined by our method are shown, and the three-pole Debye model is implemented to describe their frequency-behavior in “Permittivity measurements of sandy soil samples”. After that, permittivity-moisture curve is implemented by our method, compared with similar curves constructed by different methods in the literature, and applied for predicting moisture level of the tested sandy soil samples with different moisture levels in “Permittivity-moisture curve and prediction”. Finally, important findings are recapitulated in “Conclusion”.

## The method

**Figure 1:**
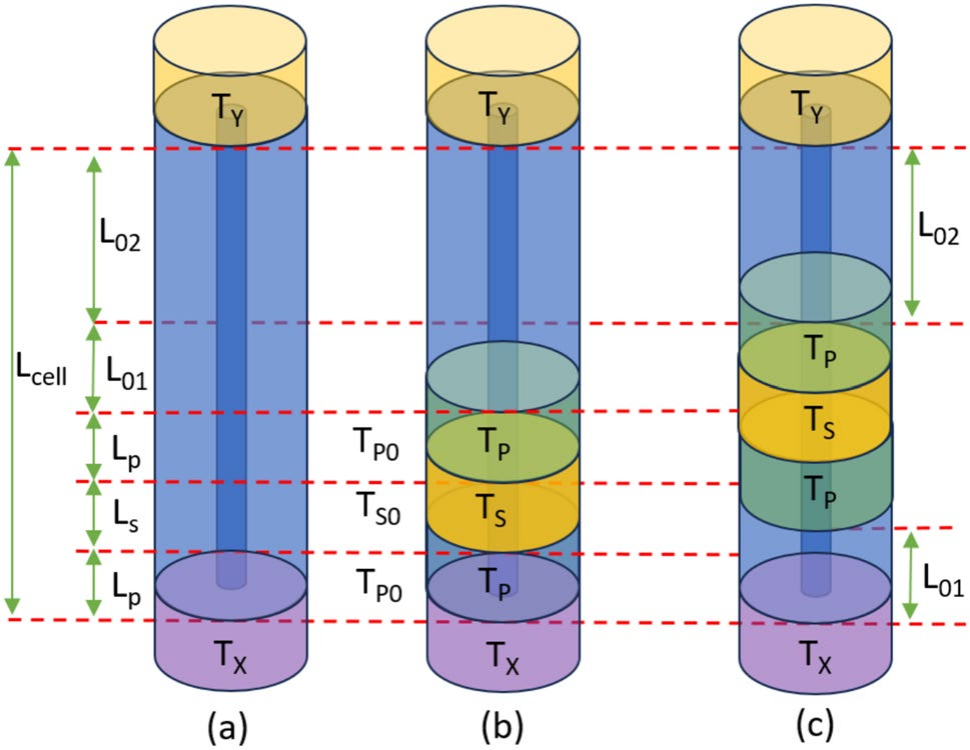
Measurement configurations: (**a**) empty hollow coaxial line with length $L_{\text {cell}}$, (**b**) the same hollow coaxial line with a sandy soil sample with length $L_s$ sandwiched between two identical plugs with lengths $L_p$, and (**c**) the same configuration in (**b**) after the plug-(sandy) soil-plug is moved upward by distance $L_{01}$.

Our proposed method utilizes the S-parameter data of three different configurations of a hollow coaxial line (e.g., EIA 1-5/8” line) with length $L_{\text {cell}}$, as shown in Fig. 1. Figure 1a illustrates the configuration of the empty line whereas Fig. 1b presents the configuration of a (sandy) soil sample with length $L_s$ and relative permittivity $\varepsilon _{rs} = \varepsilon _{rs}^{\prime } - i \varepsilon _{rs}^{\prime \prime }$, sandwiched between two identical dielectric plugs with lengths $L_p$ and relative permittivity $\varepsilon _{rp} = \varepsilon _{rp}^{\prime } - i \varepsilon _{rp}^{\prime \prime }$. Besides, Fig. 1c shows the same configuration in Fig. 1b after the plug-soil-plug structure is moved upward by a distance $L_{01}$. There are two empty air regions with lengths $L_{01}$ and $L_{02}$ inside the hollow coaxial line. In our analysis, we consider the exp$(+j \omega t)$ time reference, meaning that $\varepsilon _{rp}^{\prime \prime } \ge 0$ and $\varepsilon _{rs}^{\prime \prime } \ge 0$.

### Mathematical modeling of the configurations

The presentations of WCMs^56,57,60–62,64–66^ or TMs^26,27,67,68^ can be equally utilized for the mathematical analysis for the configurations in Fig. 1a–c. In this study, we use for the first time in the literature the combination of the STM and WCM presentations for modeling our de-embedding procedure. The STM presentation allows determination of electromagnetic properties of samples such as the propagation constant and wave impedance by directly utilizing the coupled Maxwell equations without any effort in uncoupling the complex-in-nature wave equations. According to the STM model, assuming that the propagation mode within the measurement cell is the dominant mode (TEM mode), the STM $[\Phi ]$ of the sample with $L_s$ inside a coaxial line can be written^72,73^ as

1 $$\begin{aligned} {[}\Phi ] = \begin{bmatrix}{} \Phi _{11} & 0 & 0 & \Phi _{14} \\ 0 & \Phi _{22} & \Phi _{23} & 0 \\ 0 & \Phi _{32} & \Phi _{33} & 0 \\ \Phi _{41} & 0 & 0 & \Phi _{44} \end{bmatrix}, \end{aligned}$$

where $\Phi _{11}$, $\Phi _{14}$, $\Phi _{22}$, $\Phi _{23}$, $\Phi _{32}$, $\Phi _{33}$, $\Phi _{41}$, and $\Phi _{44}$ are the non-zero state elements. Their expressions are

2 $$\begin{aligned} & \Phi _{11} = \text {cosh} (\lambda _s), \ \ \Phi _{14} = \frac{ Z }{ \sqrt{\varepsilon _{rs}} } \text {sinh} (\lambda _s), \end{aligned}$$

3 $$\begin{aligned} & \Phi _{22} = \Phi _{11}, \ \ \ \Phi _{23} = - \frac{ Z }{ \sqrt{\varepsilon _{rs}} } \text {sinh} (\lambda _s) = - \Phi _{14}, \end{aligned}$$

4 $$\begin{aligned} & \Phi _{32} = - \frac{ \sqrt{\varepsilon _{rs}} }{ Z } \text {sinh} (\lambda _s), \ \ \ \Phi _{33} = \Phi _{11}, \end{aligned}$$

5 $$\begin{aligned} & \Phi _{41} = \frac{ \sqrt{\varepsilon _{rs}} }{ Z } \text {sinh} (\lambda _s) = - \Phi _{32}, \ \ \ \Phi _{44} = \Phi _{11}. \end{aligned}$$

Here, $Z = Z_0/\sqrt{\varepsilon _{rs}} = (\eta _0/\sqrt{\varepsilon _{rs}}) \text {ln} (b/a)/(2 \pi )$ where $Z_0$ is the characteristic impedance of the empty coaxial line; *a* and *b* are, respectively, the radii of the inner and outer conductors; and $\lambda _s$ is the eigenvalue of the matrix $[A] = -[\Gamma _w] L_s$ where

6 $$\begin{aligned} \lambda _s = i k_0 \sqrt{\varepsilon _{rs}} L_s, \end{aligned}$$

and $[\Gamma _{\omega }]$ is a term associated with the state vector by

7 $$\begin{aligned} {[}\Gamma _{\omega }] = \begin{bmatrix}{} 0 & 0 & 0 & -i \omega \mu _0 \\ 0 & 0 & i \omega \mu _0 & 0 \\ 0 & i \omega \varepsilon _0 \varepsilon _{rs} & 0 & 0 \\ -i \omega \varepsilon _0 \varepsilon _{rs} & 0 & 0 & 0 \end{bmatrix}. \end{aligned}$$

Using the relation between S-parameters ($S_{11}$, $S_{21}$, $S_{12}$, and $S_{22}$) and the WCM presentation $[M_k]$

8 $$\begin{aligned} & {[} M_k ] = \frac{1}{S_{21}} \begin{bmatrix} (S_{21} S_{12} - S_{11} S_{22}) & S_{11} \\ -S_{22} & 1 \\ \end{bmatrix}, \end{aligned}$$

9 $$\begin{aligned} & S_{11} = S_{22} = \frac{ \Phi _{22} - \Phi _{33} + Z \Phi _{32} - \Phi _{23}/Z }{ \Phi _{22} + \Phi _{33} - Z \Phi _{32} - \Phi _{23}/Z }, \end{aligned}$$

10 $$\begin{aligned} & S_{21} = S_{12} = \frac{2}{\Phi _{22} + \Phi _{33} - Z \Phi _{32} - \Phi _{23}/Z}, \end{aligned}$$

one can determine the WCM of the sample with $L_s$ ($T_s$) inside a coaxial line as

11 $$\begin{aligned} {[} T_s ] = \begin{bmatrix} \Upsilon _1 & \Upsilon _2 \\ -\Upsilon _2 & \Upsilon _3 \\ \end{bmatrix}, \end{aligned}$$

where

12 $$\begin{aligned} & \Upsilon _1 = \frac{ 2 - ( \sqrt{\varepsilon _{rs}} - 1/\sqrt{\varepsilon _{rs}} )^2 \text {sinh}^2 (\lambda _s)/2 }{ 2 \text {cosh} (\lambda _s) + ( \sqrt{\varepsilon _{rs}} + 1/\sqrt{\varepsilon _{rs}} ) \text {sinh} (\lambda _s) }, \end{aligned}$$

13 $$\begin{aligned} & \Upsilon _2 = - ( \sqrt{\varepsilon _{rs}} - 1/\sqrt{\varepsilon _{rs}} ) \text {sinh} (\lambda _s)/2, \end{aligned}$$

14 $$\begin{aligned} & \Upsilon _3 = \text {cosh} (\lambda _s) + ( \sqrt{\varepsilon _{rs}} + 1/\sqrt{\varepsilon _{rs}} ) \text {sinh} (\lambda _s)/2. \end{aligned}$$

In a similar fashion, the WCM of the plug with $L_p$ inside a coaxial line can be written as

15 $$\begin{aligned} {[} T_p ] = \begin{bmatrix} \Lambda _1 & \Lambda _2 \\ -\Lambda _2 & \Lambda _3 \\ \end{bmatrix}, \end{aligned}$$

where $$\lambda _p = i k_0 \sqrt{\varepsilon _{rp}} L_p$$ and

16 $$\begin{aligned} & \Lambda _1 = \frac{ 2 - ( \sqrt{\varepsilon _{rp}} - 1/\sqrt{\varepsilon _{rp}} )^2 \text {sinh}^2 (\lambda _p)/2 }{ 2 \text {cosh} (\lambda _p) + ( \sqrt{\varepsilon _{rp}} + 1/\sqrt{\varepsilon _{rp}} ) \text {sinh} (\lambda _p) }, \end{aligned}$$

17 $$\begin{aligned} & \Lambda _2 = - ( \sqrt{\varepsilon _{rp}} - 1/\sqrt{\varepsilon _{rp}} ) \text {sinh} (\lambda _p)/2, \end{aligned}$$

18 $$\begin{aligned} & \Lambda _3 = \text {cosh} (\lambda _p) + ( \sqrt{\varepsilon _{rp}} + 1/\sqrt{\varepsilon _{rp}} ) \text {sinh} (\lambda _p)/2. \end{aligned}$$

Besides, air regions with lengths $L_p$, $L_s$, $L_{01}$, and $L_{02}$ are determined from (11)-(14) by letting $\varepsilon _{rs} = 1.0$ as

19 $$\begin{aligned} {[} T_{u0} ] = \begin{bmatrix} \alpha _u & 0 \\ 0 & 1/\alpha _u \\ \end{bmatrix}, \ \ \alpha _u = e^{- i k_0 L_u}, \end{aligned}$$

where $$u = \text {p, s, 01, or 02}$$.

### Elimination of error terms

The WCMs of the configurations in Fig. 1a-c can be expressed by

20 $$\begin{aligned} & {[}M_a] = [T_X] [T_{p0}] [T_{s0}] [T_{p0}] [T_{01}] [T_{02}] [T_Y], \end{aligned}$$

21 $$\begin{aligned} & {[}M_b] = [T_X] [T_p] [T_s] [T_p] [T_{01}] [T_{02}] [T_Y], \end{aligned}$$

22 $$\begin{aligned} & {[}M_c] = [T_X] [T_{01}] [T_p] [T_s] [T_p] [T_{02}] [T_Y], \end{aligned}$$

where $[M_a]$, $[M_b]$, and $[M_c]$ are the WCMs of the configurations in Fig. 1a–c, and $[T_X]$ and $[T_Y]$ are WCMs of the error networks *X* and *Y*. Here, $[T_X]$ and $[T_Y]$ involve systematic errors of a VNA including frequency and source/load match errors and effects of adapters and transitions, together with phase and amplitude changes arising from transitions. $[M_a]$, $[M_b]$, and $[M_c]$ are known parameters, which are associated with measured S-parameters by the relation in (8).

In addition to the measurement configurations in Fig. 1a–c, we assume that we utilize reversed configurations of those in Fig. 1b,c, having the following WCMs

23 $$\begin{aligned} & {[}M_a^{\text {rev}}] = [T_X] [T_{02}] [T_{01}] [T_{p0}] [T_{s0}] [T_{p0}] [T_Y], \end{aligned}$$

24 $$\begin{aligned} & {[}M_c^{\text {rev}}] = [T_X] [T_{02}] [T_p] [T_s] [T_p] [T_{01}] [T_Y]. \end{aligned}$$

We apply the following two-step procedure for eliminating the unknowns $[T_X]$ and $[T_Y]$ from our analysis. In the first step, we multiply one of the WCMs in (20)-(24) with another one, resulting in

25 $$\begin{aligned} & {[}M_c] [M_b]^{-1} = [T_X] [T_{01}] [T_p] [T_s] [T_p] [T_{01}]^{-1} [T_p]^{-1} [T_s]^{-1} [T_p]^{-1} [T_X]^{-1} \end{aligned}$$

26 $$\begin{aligned} & {[}M_b^{\text {rev}}] [M_c]^{-1} = [T_X] [T_{01}] [T_{02}] [T_p] [T_s] [T_p] [T_{02}]^{-1} [T_p]^{-1} [T_s]^{-1} [T_p]^{-1} [T_{01}]^{-1} [T_X]^{-1}, \end{aligned}$$

27 $$\begin{aligned} & {[}M_b^{\text {rev}}] [M_b]^{-1} = [T_X] [T_{01}] [T_{02}] [T_p] [T_s] [T_p] [T_{02}]^{-1} [T_{01}]^{-1} [T_p]^{-1} [T_s]^{-1} [T_p]^{-1} [T_X]^{-1}, \end{aligned}$$

28 $$\begin{aligned} & {[}M_c^{\text {rev}}] [M_c]^{-1} = [T_X] [T_{02}] [T_p] [T_s] [T_p] [T_{01}] [T_{02}]^{-1} [T_p]^{-1} [T_s]^{-1} [T_p]^{-1} [T_{01}]^{-1} [T_X]^{-1}, \end{aligned}$$

29 $$\begin{aligned} & {[}M_b^{\text {rev}}] [M_a]^{-1} = [T_X] [T_p] [T_s] [T_p] [T_{p0}]^{-1} [T_{s0}]^{-1} [T_{p0}]^{-1} [T_X]^{-1}. \end{aligned}$$

It is seen from (25)-(29) that the effect of $[T_Y]$ on measurements is eliminated after the first step.

As a second step, we exploit the similarity of matrices (that is, the same trace values) on the left and right sides of the expressions in (25)-(29). Application of the trace operation and incorporating the expressions $[T_s]$, $[T_p]$, $[T_{01}]$ and $[T_{02}]$ yields the following expressions

30 $$\begin{aligned} \Omega _1= & \text {Tr} ( [M_c] [M_b]^{-1} ) = \text {Tr} ([T_{01}] [T_p] [T_s] [T_p] [T_{01}]^{-1} [T_{p0}]^{-1} [T_{s0}]^{-1} [T_{p0}]^{-1} ) \nonumber \\= & 2 \Psi _1 \Psi _3 + (\alpha _{01}^2 + \frac{1}{\alpha _{01}^2}) \Psi _2^2, \end{aligned}$$

31 $$\begin{aligned} \Omega _2= & \text {Tr} ( [M_b^{\text {rev}}] [M_c]^{-1} ) = \text {Tr} ([T_{02}] [T_p] [T_s] [T_p] [T_{02}]^{-1} [T_{p0}]^{-1} [T_{s0}]^{-1} [T_{p0}]^{-1} ) \nonumber \\= & 2 \Psi _1 \Psi _3 + (\alpha _{02}^2 + \frac{1}{\alpha _{02}^2}) \Psi _2^2, \end{aligned}$$

32 $$\begin{aligned} \Omega _3= & \text {Tr} ( [M_b^{\text {rev}}] [M_b]^{-1} ) = \text {Tr} ([T_{01}] [T_{02}] [T_p] [T_s] [T_p] [T_{02}]^{-1} [T_{01}]^{-1} [T_{p0}]^{-1} [T_{s0}]^{-1} [T_{p0}]^{-1} ) \nonumber \\= & 2 \Psi _1 \Psi _3 + (\alpha _{01}^2 \alpha _{02}^2 + \frac{1}{\alpha _{01}^2 \alpha _{02}^2}) \Psi _2^2, \end{aligned}$$

33 $$\begin{aligned} \Omega _4= & \text {Tr} ( [M_c^{\text {rev}}] [M_c]^{-1} ) = \text {Tr} ([T_{02}] [T_p] [T_s] [T_p] [T_{01}] [T_{02}]^{-1} [T_{p0}]^{-1} [T_{s0}]^{-1} [T_{p0}]^{-1} [T_{01}]^{-1}) \nonumber \\= & 2 \Psi _1 \Psi _3 + (\frac{\alpha _{01}^2}{\alpha _{02}^2} + \frac{\alpha _{02}^2}{\alpha _{01}^2}) \Psi _2^2, \end{aligned}$$

where

34 $$\begin{aligned} & \Psi _1 = \dfrac{ 2 (\Lambda _1^2 - \Lambda _2^2) \sqrt{\varepsilon _{rs}} \text {cosh} (\lambda _s) - \Big [ (\Lambda _1 - \Lambda _2)^2 \varepsilon _{rs} + (\Lambda _1 + \Lambda _2)^2 \Big ] \text {sinh} (\lambda _s) }{ 2 \sqrt{\varepsilon _{rs}} }, \end{aligned}$$

35 $$\begin{aligned} & \Psi _2 = \dfrac{ 2 \Lambda _2 (\Lambda _1 + \Lambda _3) \sqrt{\varepsilon _{rs}} \text {cosh} (\lambda _s) + [ \Lambda _2 (\Lambda _3 - \Lambda _1) (1 + \varepsilon _{rs}) + (\Lambda _1 \Lambda _3 - \Lambda _2^2) (1 - \varepsilon _{rs} ) ] \text {sinh} (\lambda _s) }{ 2 \sqrt{\varepsilon _{rs}} }, \end{aligned}$$

36 $$\begin{aligned} & \Psi _3 = \dfrac{ 2 (\Lambda _3^2 - \Lambda _2^2) \sqrt{\varepsilon _{rs}} \text {cosh} (\lambda _s) + \Big [ (\Lambda _2 + \Lambda _3)^2 \varepsilon _{rs} + (\Lambda _2 - \Lambda _3)^2 \Big ] \text {sinh} (\lambda _s) }{ 2 \sqrt{\varepsilon _{rs}} }, \end{aligned}$$

It is seen from (30)-(36) that the effect of the other unknown parameter $[T_X]$ on measurements is removed.

### Extraction procedure

Our goal here is to determine $\varepsilon _{rs}$ with no knowledge of $L_{01}$, $L_{02}$, and $L_s$ using the expressions in (30)-(36). From (30)-(33), it is possible to eliminate the product $\Psi _1 \Psi _3$ involving the unknown $\varepsilon _{rs}$ by the following expressions

37 $$\begin{aligned} & \Omega _1 - \Omega _2 = \bigg [ \left( \alpha _{01}^2 + \frac{1}{\alpha _{01}^2} \right) - \left( \alpha _{02}^2 + \frac{1}{\alpha _{02}^2} \right) \bigg ] \Psi _2^2, \end{aligned}$$

38 $$\begin{aligned} & \Omega _1 - \Omega _3 = \bigg [ \left( \alpha _{01}^2 + \frac{1}{\alpha _{01}^2} \right) - \left( \alpha _{01}^2 \alpha _{02}^2 + \frac{1}{\alpha _{01}^2 \alpha _{02}^2} \right) \bigg ] \Psi _2^2, \end{aligned}$$

39 $$\begin{aligned} & \Omega _1 - \Omega _4 = \bigg [ \left( \alpha _{01}^2 + \frac{1}{\alpha _{01}^2} \right) - \left( \frac{\alpha _{02}^2}{\alpha _{01}^2} + \frac{\alpha _{01}^2}{\alpha _{02}^2} \right) \bigg ] \Psi _2^2, \end{aligned}$$

It is further possible to remove the $\varepsilon _{rs}$ dependence through (37)-(39) by eliminating $\Psi _2^2$ term

40 $$\begin{aligned} & \frac{\Omega _1 - \Omega _2}{\Omega _1 - \Omega _3} = \zeta _1 = \dfrac{ \left( \alpha _{01}^2 + \frac{1}{\alpha _{01}^2} \right) - \left( \alpha _{02}^2 + \frac{1}{\alpha _{02}^2} \right) }{ \left( 1 - \alpha _{02}^2 \right) \alpha _{01}^2 + \left( 1 - \frac{1}{\alpha _{02}^2} \right) \frac{1}{\alpha _{01}^2} }, \end{aligned}$$

41 $$\begin{aligned} & \frac{\Omega _1 - \Omega _2}{\Omega _1 - \Omega _4} = \zeta _2 = \dfrac{ \left( \alpha _{01}^2 + \frac{1}{\alpha _{01}^2} \right) - \left( \alpha _{02}^2 + \frac{1}{\alpha _{02}^2} \right) }{ \left( 1 - \frac{1}{\alpha _{02}^2} \right) \alpha _{01}^2 + \left( 1 - \alpha _{02}^2 \right) \frac{1}{\alpha _{01}^2} }, \end{aligned}$$

Utilizing the expressions in (40) and (41) simultaneously, one can determine $\alpha _{01}^2$ in terms of $\alpha _{02}^2$ as

42 $$\begin{aligned} \alpha _{01}^2 = \dfrac{ \dfrac{1/\alpha _{02}^2}{ 1 - \zeta _2 (1 - \alpha _{02}^2) } - \dfrac{1}{ \alpha _{02}^2 + \zeta _1 (1 - \alpha _{02}^2) } }{ \dfrac{\zeta _1 (1 - \alpha _{02}^2) - 1}{ \alpha _{02}^2 + \zeta _1 (1 - \alpha _{02}^2) } - \dfrac{\zeta _2 (1 - 1/\alpha _{02}^2) - 1}{ \alpha _{02}^2 [1 - \zeta _2 (1 - \alpha _{02}^2) ] } }. \end{aligned}$$

Therefore, incorporating $\alpha _{01}^2$ into either (40) or (41), one can determine $L_{02}$, to be demonstrated in “Numerical and accuracy analyses”, by means of the following objective functions

43 $$\begin{aligned} & F_1 ( L_{02} ) = \left| \zeta _1 - \dfrac{ \left( \alpha _{01}^2 + \frac{1}{\alpha _{01}^2} \right) - \left( \alpha _{02}^2 + \frac{1}{\alpha _{02}^2} \right) }{ \left( 1 - \alpha _{02}^2 \right) \alpha _{01}^2 + \left( 1 - \frac{1}{\alpha _{02}^2} \right) \frac{1}{\alpha _{01}^2} } \right| = 0, \end{aligned}$$

44 $$\begin{aligned} & F_2 ( L_{02} ) = \left| \zeta _2 - \dfrac{ \left( \alpha _{01}^2 + \frac{1}{\alpha _{01}^2} \right) - \left( \alpha _{02}^2 + \frac{1}{\alpha _{02}^2} \right) }{ \left( 1 - \frac{1}{\alpha _{02}^2} \right) \alpha _{01}^2 + \left( 1 - \alpha _{02}^2 \right) \frac{1}{\alpha _{01}^2} } \right| = 0. \end{aligned}$$

where $|\star |$ denotes the magnitudes of the complex quantity ‘$\star$’.

Afterwards, $L_{01}$ can be calculated from (see “Numerical and accuracy analyses”)

45 $$\begin{aligned}&G ( L_{01} ) = 0 = \left| \alpha _{01}^2 - \dfrac{ \dfrac{1/\alpha _{02}^2}{ 1 - \zeta _2 (1 - \alpha _{02}^2) } - \dfrac{1}{ \alpha _{02}^2 + \zeta _1 (1 - \alpha _{02}^2) } }{ \dfrac{\zeta _1 (1 - \alpha _{02}^2) - 1}{ \alpha _{02}^2 + \zeta _1 (1 - \alpha _{02}^2) } - \dfrac{\zeta _2 (1 - 1/\alpha _{02}^2) - 1}{ \alpha _{02}^2 [1 - \zeta _2 (1 - \alpha _{02}^2) ] } } \right| . \end{aligned}$$

Then, $L_s$ can be found from

46 $$\begin{aligned} L_s = L_{\text {cell}} - 2 L_p - L_{01} - L_{02}, \end{aligned}$$

using computed $L_{01}$ from (43) or (44) and $L_{02}$ from (45) for known $L_p$ and $L_{\text {cell}}$ values.

Besides, from (29), one can obtain

47 $$\begin{aligned} \text {Tr} ([M_b^{\text {rev}}] [M_a]^{-1}) = \Omega _5 = \frac{1}{\alpha _{0p}^2 \alpha _{0s}} \Psi _1 + \alpha _{0p}^2 \alpha _{0s} \Psi _3, \end{aligned}$$

where $\Psi _1$ and $\Psi _3$ are presented in (34) and (36), respectively.

Finally, as to be shown in “Numerical and accuracy analyses”, $\varepsilon _{rs}$ can be uniquely determined by way of a suitable 2D numerical method using the following metric functions

48 $$\begin{aligned}&H_1 (\varepsilon _{rs}) = 0 = \left| \Re e \left\{ \Omega _5 \frac{4 \sqrt{\varepsilon _{rs}} }{ (1 + \sqrt{\varepsilon _{rs}} )^2 } e^{- \lambda _s} - \frac{\Psi _1}{ \alpha _{0p}^2 \alpha _{0s}} - \alpha _{0p}^2 \alpha _{0s} \Psi _3 \right\} \right| , \end{aligned}$$

49 $$\begin{aligned}&H_2 (\varepsilon _{rs}) = 0 = \left| \Im m \left\{ \Omega _5 \frac{4 \sqrt{\varepsilon _{rs}} }{ (1 + \sqrt{\varepsilon _{rs}} )^2 } e^{- \lambda _s} - \frac{\Psi _1}{ \alpha _{0p}^2 \alpha _{0s}} - \alpha _{0p}^2 \alpha _{0s} \Psi _3 \right\} \right| , \end{aligned}$$

where $\Re e\{ \star \}$ and $\Im m\{ \star \}$ take, respectively, the real and imaginary parts of the complex quantity ‘$\star$’.

**Figure 2:**
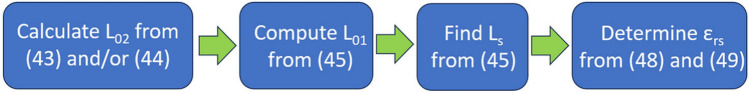
Flowchart of the proposed method to extract $\varepsilon _{rs}$.

We present the flowchart of our proposed meniscus-free extraction procedure in Fig. 2 for its implementation.

## Numerical and accuracy analyses

Here, our goal is to first examine the possibility of one solution for $L_{01}$ and $L_{02}$ and then evaluate whether unique solution could be determined for $\varepsilon _{rs}$ from the application of metric functions $H_1 (\varepsilon _{rs})$ and $H_2 (\varepsilon _{rs})$ in (48) and (49). To achieve our goal, unlike previous studies^26,27^ which are based on simulations, we implemented a numerical analysis for simplicity. Then, we synthesized electrical properties of the sample and plug by the following parameters (arbitrarily selected): $\varepsilon _{rs} = 6.225 - i 0.100$, $\varepsilon _{rp} = 2.120 - i 0.050$, $L_s = 10.0$ mm, and $L_p = 15.0$ mm. Besides, error matrices $[T_X]$ and $[T_Y]$ were assumed to be (chosen arbitrarily)

50 $$\begin{aligned} & {[}T_X] = \begin{bmatrix} 0.1 + i 0.2 & 0.5 + i 0.4 \\ 0.2 - i 0.6 & -0.3 + i 0.2 \\ \end{bmatrix}, \end{aligned}$$

51 $$\begin{aligned} & {[}T_Y] = \begin{bmatrix} 0.1 - i 0.4 & -0.2 + i 0.4 \\ -0.2 - i 0.2 & -0.7 - i 0.1 \\ \end{bmatrix}. \end{aligned}$$

**Figure 3:**
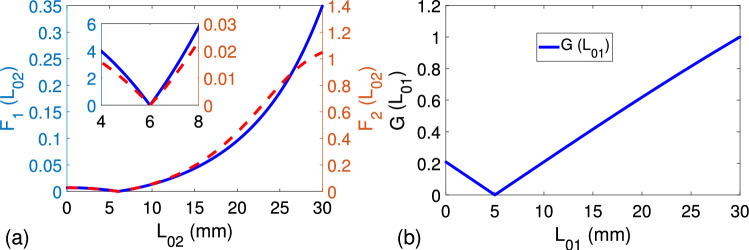
Dependence of (**a**) $F_1 (L_{02})$ in (43) and $F_2 (L_{02})$ (44) versus $L_{02}$ (mm) and (**b**) $G (L_{01})$ in (45).

Figure 3a,b demonstrate, respectively, the dependencies of $F_1 (L_{02})$, $F_2 (L_{02})$, and $G (L_{01})$ for $f = 1.0$ GHz, $L_{01} = 5.0$ mm, and $L_{02} = 6.0$ mm ($L_{\text {cell}} = 36.0$ mm). It is noted from the results in Fig. 3a that $F_1 (L_{02})$ or $F_2 (L_{02})$ produce accurate $L_{02}$ value ($\cong 6.00$ mm), indicating that either of them is sufficient to determine one solution for $L_{02}$. Besides, it is observed from the result in Fig. 3b that it is possible to compute one and accurate value of $L_{01}$ ($\cong 5.00$ mm) using the metric function $G (L_{01})$.

**Figure 4:**
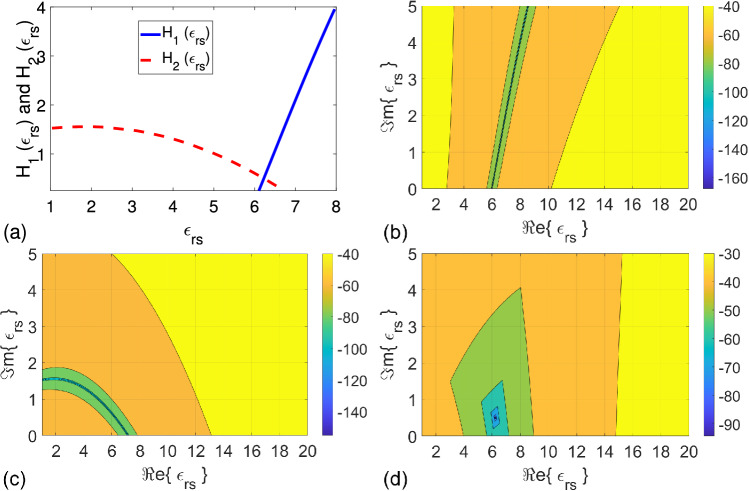
(**a**) Constant-value curves $H_1 (\varepsilon _{rs})$ in (48) and $H_2 (\varepsilon _{rs})$ in (49) versus $\varepsilon _{rs}$, (**b**) the dependence of $20 \text {log}_{10} H_1 (\varepsilon _{rs})$ over the complex $\varepsilon _{rs}$ plane, (**c**) the dependence of $20 \text {log}_{10} H_2 (\varepsilon _{rs})$ over the complex $\varepsilon _{rs}$ plane, and (**d**) the dependence of $20 \text {log}_{10} ( H_1 (\varepsilon _{rs}) + H_2 (\varepsilon _{rs}))$ over the complex $\varepsilon _{rs}$ plane.

We also implemented the metric functions $H_1 (\varepsilon _{rs})$ and $H_2 (\varepsilon _{rs})$ to access whether they are sufficient to determine one value for $\varepsilon _{rs}$. For example, Fig. 4a shows the dependencies of $H_1 (\varepsilon _{rs})$ and $H_2 (\varepsilon _{rs})$ (constant-value curves) for the case that $H_1 (\varepsilon _{rs}) \le 10^{-8}$ and $H_2 (\varepsilon _{rs}) \le 10^{-8}$. This is because it is not feasible to perfectly satisfy the null value for $H_1 (\varepsilon _{rs})$ and $H_2 (\varepsilon _{rs})$. It is seen from the results in Fig. 4a that while $H_1 (\varepsilon _{rs})$ or $H_2 (\varepsilon _{rs})$ is itself not capable of resulting in one value for $\varepsilon _{rs}$, their intersection exactly produces correct and one value for $\varepsilon _{rs}$ ($\cong 6.225 - i 0.100$). In order to examine the variation of possible values in constant-value curves around the solution region ($\cong 6.225 - i 0.100$), we obtained the dependencies of $20 \text {log}_{10} H_1 (\varepsilon _{rs})$, $20 \text {log}_{10} H_2 (\varepsilon _{rs})$, and $20 \text {log}_{10}( H_1 (\varepsilon _{rs}) + H_2 (\varepsilon _{rs}) )$ on the complex $\varepsilon _{rs}$ plane, as shown in Fig. 4b–d. It is observed from the results that are presented by color bars in Fig. 4b–d that while $20 \text {log}_{10} H_1 (\varepsilon _{rs})$ and $20 \text {log}_{10} H_2 (\varepsilon _{rs})$ change fast around the solution region, $20 \text {log}_{10}( H_1 (\varepsilon _{rs}) + H_2 (\varepsilon _{rs}) )$ varies relatively less around the solution region, manifesting that $H_1 (\varepsilon _{rs})$ and $H_2 (\varepsilon _{rs})$ should be individually analyzed and their intersection can then be implemented for unique and accurate $\varepsilon _{rs}$ determination.

## Measurement setup and validation

**Figure 5:**
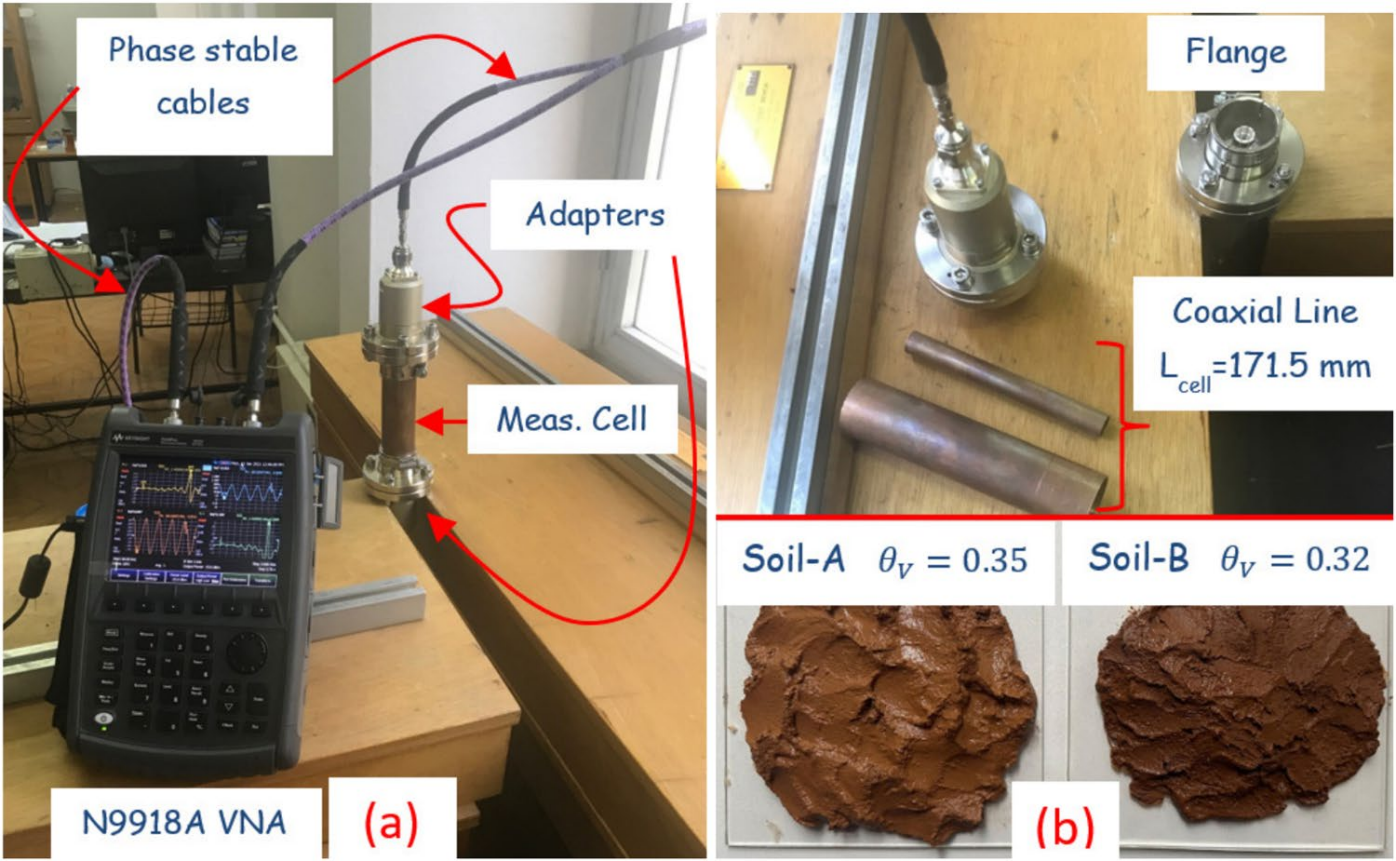
Pictures of (**a**) the measurement setup and (**b**) the measurement cell (inner and outer conductors) along with Sol-A with $\theta _V = 0.35$ and Soil-B with $\theta _V = 0.32$.

We performed some measurements to validate our proposed method before starting to permittivity measurements of sandy soil samples. The measurement setup, as presented in Fig. 5a, involves a vector network analyzer (VNA) purchased from Keysight Technologies (with model N9918A), two relatively flexible cables (3.5 mm), two Type-N-to-EIA 1-5/8” adapters, and a hollow coaxial line segment with length $L_{\text {cell}} = 150 \mp 0.040$ mm. This VNA sweeps S-parameters between 30 kHz and 26.5 GHz with nearly 90 dB dynamic range and directivity over 32 dB. The coaxial cables are in 100 mm long providing phase-stable measurements. The cell has an inner conductor with a diameter of $16.9 \mp 0.050$ mm and outer conductor with a diameter of $38.8 \mp 0.075$ mm^19,20,23,25–27^, as shown in Fig. 5b. Two identical polyethylene (PE) materials with length $L_p = 3.85 \mp 0.030$ mm were in charge of plugs used to eliminate the meniscus formation. It is noteworthy that there is no standardized calibration standard or procedure available for performing calibration of EIA 1-5/8” transmission lines at microwave frequencies. Therefore, our proposed method can effectively be utilized for such transmission lines^19,55^ without any calibration procedure.

**Figure 6:**
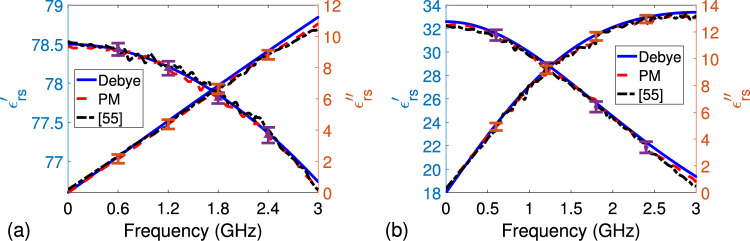
Extracted $\varepsilon _{rs}^{\prime } \text { and } \varepsilon _{rs}^{\prime \prime }$ of (**a**) distilled water and (**b**) methanol by our proposed method (PM) and the method in the study^55^ (including error bars at few discrete frequencies). Predicted $\varepsilon _{rs}^{\prime } \text { and } \varepsilon _{rs}^{\prime \prime }$ by the one-pole Debye model are also given for comparison.

Figure 6a,b illustrate extracted $\varepsilon _{rs}^{\prime }$ and $\varepsilon _{rs}^{\prime \prime }$ of distilled water and methanol samples (approximately 30 mm) by our proposed method (using average of five independent measurements) over 0.05-3.0 GHz (including error bars at few discrete frequencies). The maximum frequency was set due to the presence of higher order modes beyond 3.0 GHz for Type-N-to-EIA 1-5/8” coaxial lines. For comparison, $\varepsilon _{rs}^{\prime }$ and $\varepsilon _{rs}^{\prime \prime }$ synthesized by the one-pole Debye model, which is applicable for liquid samples, are also presented in these figures. According to the Debye model, $\varepsilon _{rs} (\omega )$ can be synthesized by

52 $$\begin{aligned} \varepsilon _{rs} (\omega ) = \varepsilon _{\infty } + \frac{\varepsilon _{s} - \varepsilon _{\infty }}{ 1 + j \omega \tau } \end{aligned}$$

where $\varepsilon _{s}$ and $\varepsilon _{\infty }$ are the permittivities at zero-frequency and infinite-frequency; and $\tau$ is the relaxation time. The dispersive model parameters used in the synthesis process are $\varepsilon _s = 78.5$, $\varepsilon _{\infty } = 5.2$, and $\tau = 8.33$ ps for the distilled water, and $\varepsilon _s = 32.59$, $\varepsilon _{\infty } = 5.65$, and $\tau = 52.10$ ps for the methanol^74^. Besides, the method in our recent study^55^ was also applied for demonstration of the effect of meniscus. We did not apply the methods in the studies^63,67,68^ because it is difficult to locate plugs at the same places of one line with air and sample fillings and because thru connection using Type-N-to-EIA 1-5/8” coaxial lines is difficult to implement. Using two identical lines with different lengths could be a remedy for the method in the study^67^; however, it will then have the same problem (plug location) of the methods in the studies^63,68^. Furthermore, the method in the study^71^ requires three different sample fillings, which are partly infeasible to implement. Besides, the method in the study^71^ has a limited accuracy for distilled water for frequencies up to 5 GHz.

It is seen from the results in Fig. 6a,b that extracted $\varepsilon _{rs}^{\prime }$ and $\varepsilon _{rs}^{\prime \prime }$ of distilled water and methanol samples by our proposed are in complete agreement with those synthesized by the one-pole Debye model over the entire frequency band. There is less than 5% difference between extracted $\varepsilon _{rs}^{\prime }$ (and $\varepsilon _{rs}^{\prime \prime }$) values by our method (and the method in the study^55^) and synthesized $\varepsilon _{rs}^{\prime }$ (and $\varepsilon _{rs}^{\prime \prime }$) values for both distilled water and methanol samples, validating the proposed extraction algorithm. Besides, it is seen from the results in Fig. 6a,b that extracted $\varepsilon _{rs}^{\prime }$ and $\varepsilon _{rs}^{\prime \prime }$ of distilled water and methanol samples by our proposed have a more smooth behavior with frequency than those extracted by the method in the study^55^, mainly due to the formation of meniscus on the surface of the test liquid samples.

## Permittivity measurements of sandy soil samples

**Figure 7:**
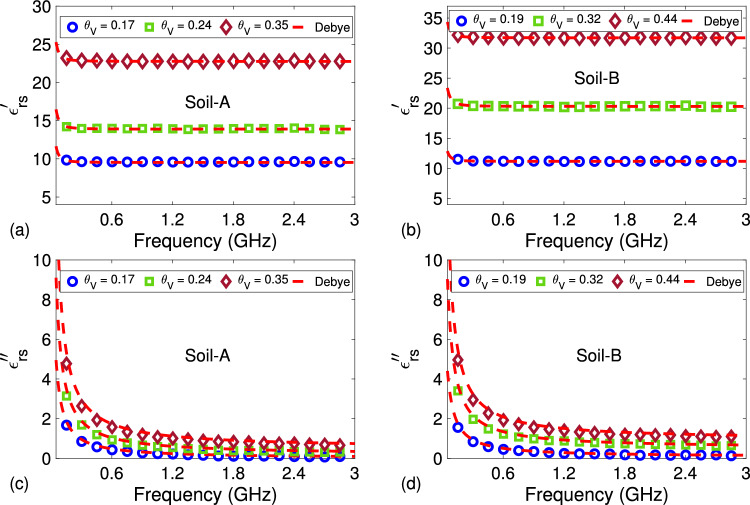
Frequency dependence of measured (denoted by blue circle, green square, and brown diamond symbols) and fitted by the three-pole Debye model (shown by dashed red lines) (**a**) $\varepsilon _{rs}^{\prime }$ and (**b**) $\varepsilon _{rs}^{\prime \prime }$ for Soil-A ($\theta _V = 0.17$, 0.24, and 0.35), and (**c**) $\varepsilon _{rs}^{\prime }$ and (**d**) $\varepsilon _{rs}^{\prime \prime }$ for Soil-B ($\theta _V = 0.19$, 0.32, and 0.44) (averaged by five independent measurements).

Measurements of $\varepsilon _{rs}^{\prime }$ and $\varepsilon _{rs}^{\prime \prime }$ were performed for two different sandy soil samples (labeled by Soil-A and Soil-B) with different volumetric water content $\theta _V$ values. Soils having more than 90% sand content were extracted from different areas of the city Gaziantep in Türkiye. While Soil-A has $\theta _V = 0.17$, 0.24, and 0.35, Soil-B has $\theta _V = 0.19$, 0.32, and 0.44. In preparation of these samples, we paid special attention on their workability ($0.17 \le \theta _V \le 0.44$) and mixing time (more than 3 minutes of slow-mixing) so that water was utterly absorbed by these soil samples to have sufficient homogeneity. Figure 5b demonstrates a photo of the Soil-A with $\theta _V= 0.35$ and Soil-B $\theta _V= 0.32$. Besides, we were also careful in positioning prepared samples inside the hollow coaxial line by pouring a small amount each time on the lower plug and by slightly tilting each sandy soil sample before pressing the upper plug relatively deeply to perform permittivity measurements. Figure 7a–d illustrate extracted $\varepsilon _{rs}^{\prime }$ and $\varepsilon _{rs}^{\prime \prime }$ of these sandy soil samples (25 mm $\le L_s \le 45$ mm) with various $\theta _V$ values (designated by circle, square, and diamond symbols) by the proposed method using the average of five independent S-parameter measurements for each configuration in Fig. 1a–c and their inverses ($L_{01} \cong 12.32$ mm). It is noted that inverse configurations were simply realized by switching port connections to the line. It is noted that slow and uniform pouring of tested sandy soil samples was first exercised, and sufficient press on the top plug was then applied for each sandy soil sample between the plugs in Fig. 1b,c to eliminate the effect of possible air bubbles and have a relatively homogeneous soil distribution over the length of the line^26,27^. Since standard deviations of extracted $\varepsilon _{rs}^{\prime }$ and $\varepsilon _{rs}^{\prime \prime }$ for Soil-A with $\theta _V = 0.17$, 0.24, and 0.35 and Soil-B with $\theta _V = 0.19$, 0.32, and 0.44 were observed to be less than approximately 0.25, their values are not shown in Fig. 7a–d for better view. However, the effect of these values on the construction of soil-moisture calibration curve will be discussed in “Permittivity-moisture curve and prediction”.

In order to describe the dispersion behavior of the extracted $\varepsilon _{rs}^{\prime }$ and $\varepsilon _{rs}^{\prime \prime }$ of Soil-A and Soil-B with different $\theta _V$ values, we also applied the Debye model. To achieve better synthesis by this model, we implemented the three-pole Debye model for each sandy soil sample^20,27^

53 $$\begin{aligned} \varepsilon _{rs} (\omega ) = \varepsilon _{\infty } - j \frac{\sigma _b}{ \omega \varepsilon _0 } + \sum _{u = 1}^{3} \frac{\Delta \varepsilon _u}{ 1 + j \omega \tau _u }, \end{aligned}$$

where $\sigma _b$ is the direct-current (zero frequency) bulk electrical conductivity; and $\Delta \varepsilon _u$ and $\tau _u$ denote the amplitude and time of relaxation for the *u*th pole. The ‘cftool’ function of MATLAB was implemented to determine these parameters using a ‘custom equation’ tab involving the parameters in (53). Here, minimum values of these parameters were set to be greater than zero to have meaningful results for $\varepsilon _{\infty }$, $\Delta \varepsilon _1$, $\Delta \varepsilon _2$, $\Delta \varepsilon _3$, $\tau _1$, $\tau _2$, and $\tau _3$.

**Table 2:** Debye parameters of the tested sandy soil samples calculated from measured data by the proposed method (PM) and the method in the study^55^.

Method	Sample	$\theta _V$	$\varepsilon _{\infty }$	$\Delta \varepsilon _1$	$\Delta \varepsilon _2$	$\Delta \varepsilon _3$	$\tau _1$	$\tau _2$	$\tau _3$	$R^2$
PM	Soil-A	0.17	9.525	5.267	4.613	3.213	8.424	7.011	6.317	0.9229
		0.24	13.897	5.541	4.811	3.076	7.286	6.785	5.384	0.9212
		0.35	22.767	6.851	5.126	4.585	8.876	7.961	6.007	0.9323
	Soil-B	0.19	10.167	5.381	3.844	1.513	8.340	7.016	5.994	0.9313
		0.32	20.306	4.883	3.581	3.396	6.206	5.818	4.327	0.9325
		0.44	29.289	5.531	4.653	3.765	8.332	6.658	5.218	0.9296
^55^	Soil-A	0.17	9.435	5.133	4.547	3.011	8.524	7.118	6.525	0.9176
		0.24	14.330	5.329	4.562	3.174	7.030	6.285	5.084	0.9266
		0.35	22.150	6.050	5.526	4.085	8.600	7.660	6.322	0.9118
	Soil-B	0.19	10.610	5.983	3.439	1.439	7.640	6.389	5.266	0.9317
		0.32	20.410	4.632	3.689	3.112	6.270	5.755	4.697	0.9107
		0.44	31.712	5.386	4.204	3.316	8.530	6.773	5.471	0.8964

Dependencies of $\varepsilon _{rs}^{\prime }$ and $\varepsilon _{rs}^{\prime \prime }$ synthesized by this model are presented by the red dashed lines in Fig. 7a–d. Calculated $\varepsilon _{\infty }$, $\Delta \varepsilon _u$, and $\tau _u$ values ($\sigma _b \le 0.0015$) for our proposed method and the method in the study^55^ are given in Table 2. It is noted that the relaxation time, which indicates how fast a medium returns to its equilibrium state after being disturbed by an external electromagnetic stimulus, of materials at microwave frequencies in general is function of electrical conductivity, Maxwell-Wagner polarization, and microscopically confined water. For soil samples, in addition to above factors, the relaxation time also depends on geometry, clay content, water structure, and macro- or micro-scale confinement in a complicated manner^75–77^. In general, a soil sample with a higher relaxation time behaves as an ‘viscoelastic’ medium with a slower pore water pressure dissipation and thereby slower consolidation^75^. It is noteworthy from the results in Table 2 that for Soil-A and Soil-B (sandy) soil samples, relaxation times $\tau _1$, $\tau _2$, and $\tau _3$ have a slight decrease for an increase of $\theta _V$ from 0.17 to 0.24 (Soil-A) and from 0.19 to 0.32 (Soil-B). This can be explained by the effective role in sandy soil samples of increased water content which reduces interparticle frictions between particles, resulting a faster redistribution of an externally applied stress. On the other hand, Soil-A sample with $\theta _V = 0.35$ and Soil-B sample with $\theta _V = 0.44$ can be considered as saturated soil samples with an increased relaxation time (slow relaxation) due to viscous flow of pore water. Figure 7a–d show fitted $\varepsilon _{rs}^{\prime }$ values (denoted by ‘Model’) versus frequency. It is seen from Fig. 7a–d that $R^2$ values, a statistical measure indicating how much a dependent variable is specified by an independent variable or independent variables in a regression model, are above approximately 0.90. Therefore, it can be stated that fitted and measured $\varepsilon _{rs}^{\prime }$ values are in good agreement for each $\theta _V$ content of any tested sandy soil sample, meaning that fitted $\varepsilon _{rs}^{\prime }$ values could be used to construct a permittivity-moisture curve, to be discussed presently.

**Figure 8:**
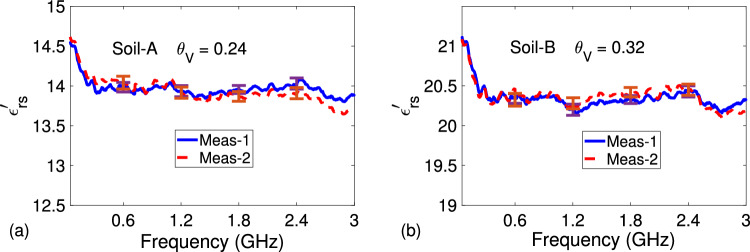
Frequency dependence of measured $\varepsilon _{rs}^{\prime }$ (**a**) of Soil-A with $\theta _V = 0.24$ for two different lengths and (**b**) of Soil-B with $\theta _V = 0.32$ for two different lengths (averaged by five independent measurements including error bars at few discrete frequencies).

**Figure 9:**
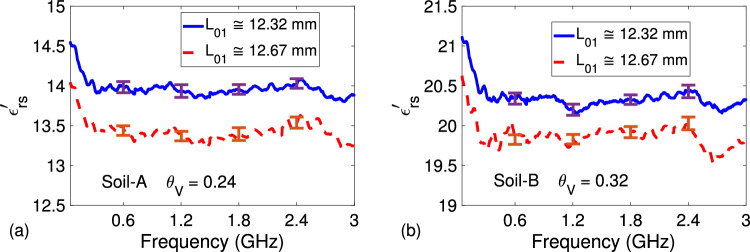
Frequency dependence of measured (**a**) $\varepsilon _{rs}^{\prime }$ of Soil-A with $\theta _V = 0.24$ for two different lengths and (**b**) $\varepsilon _{rs}^{\prime }$ of Soil-B with $\theta _V = 0.32$ for two different lengths (averaged by five independent measurements including error bars at few discrete frequencies).

Finally, we performed additional permittivity measurements to validate thickness-independent feature of our extraction procedure and its dependence $L_{01}$ value. We mainly focus on the $\varepsilon _{rs}^{\prime }$ parameter because this is the parameter directly utilized in the construction of permittivity-moisture curve, to be discussed in “Permittivity-moisture curve and prediction”. Figure 8a,b demonstrate extracted $\varepsilon _{rs}^{\prime }$ of Soil-A with $\theta _V = 0.24$ and Soil-B with $\theta _V = 0.32$ for two different thicknesses between 25 mm $\le L_s \le 45$ mm. It is seen from the results in Fig. 8a,b that extracted $\varepsilon _{rs}^{\prime }$ values for either sandy soil sample with different lengths have a maximum difference of $0.92\%$ over the entire frequency band, indicating thickness-independent feature of our extraction procedure. Besides, Fig. 9a,b illustrate the frequency dependence of the $\varepsilon _{rs}^{\prime }$ parameter of Soil-A with $\theta _V = 0.24$ and Soil-B with $\theta _V = 0.32$ extracted by the proposed method when there is a $+3\%$ offset (12.67 mm) from the actual $L_{01}$ value (12.32 mm). It is seen from Fig. 9a,b that the values of extracted $\varepsilon _{rs}^{\prime }$ by the proposed method, in general, lower down by approximately $4\%$ for both sandy soil samples when the actual $L_{01}$ value is reduced by $3\%$, indicating the importance of accurate $L_{01}$ information for precise $\varepsilon _{rs}^{\prime }$ determination by the proposed method.

## Permittivity-moisture curve and prediction

Since a permittivity-moisture curve relies on extracted $\varepsilon _{rs}^{\prime }$ only^20,27,55,78–80^, we implemented a conformity assessment for extracted $\varepsilon _{rs}^{\prime }$ by our proposed method with the Mironov-Fomin model^81,82^. This model relates $\varepsilon _{rs}^{\prime }$ to $\theta _V$ by^81,82^

54 $$\begin{aligned} \varepsilon _{rs}^{\prime } = n_s^2 - \kappa _s^2, \end{aligned}$$

where

55 $$\begin{aligned} & n_s = {\left\{ \begin{array}{ll} n_d + (n_b - 1) \theta _V, & \theta _V \le W_t \\ n_d + (n_b - 1) W_t + (n_u - 1) (\theta _V - W_t), & \theta _V \ge W_t \end{array}\right. } \end{aligned}$$

56 $$\begin{aligned} & k_s = {\left\{ \begin{array}{ll} k_d + (k_b - 1) \theta _V, & \theta _V \le W_t \\ k_d + (k_b - 1) W_t + (k_u - 1) (\theta _V - W_t), & \theta _V \ge W_t \end{array}\right. } \end{aligned}$$

and

57 $$\begin{aligned} & W_t = 0.0286 + 0.00307 C, \end{aligned}$$

58 $$\begin{aligned} & n_d = 1.634 - 0.00539C + 2.75 \cdot 10^{-5} C^2, \end{aligned}$$

59 $$\begin{aligned} & k_d = 0.0395 - 4.038 \cdot 10^{-4} C, \end{aligned}$$

60 $$\begin{aligned} & n_b = (8.86 + 0.00321 T) + (-0.0644 + 7.96 \cdot 10^{-4} T) C + (2.97 \cdot 10^{-4} - 9.6 \cdot 10^{-6} T) C^2, \end{aligned}$$

61 $$\begin{aligned} & k_b = (0.738 - 0.00903 T + 8.57 \cdot 10^{-5} T^2) + (-0.00215 + 1.47 \cdot 10^{-4} T) C \nonumber \\ & + (7.36 \cdot 10^{-5} - 1.03 \cdot 10^{-6} T + 1.05 \cdot 10^{-8} T^2 ) C^2, \end{aligned}$$

62 $$\begin{aligned} & n_u = (10.3 - 0.0173 T) + (6.5 \cdot 10^{-4} + 8.82 \cdot 10^{-5} T) C - ( 6.34 \cdot 10^{-6} + 6.32 \cdot 10^{-7} T) C^2, \end{aligned}$$

63 $$\begin{aligned} & k_u = (0.7 - 0.017 T + 1.78 \cdot 10^{-4} T^2) + (0.0161 + 7.25 \cdot 10^{-4} T) C \nonumber \\ & - (1.46 \cdot 10^{-4} + 6.03 \cdot 10^{-6} T + 7.87 \cdot 10^{-9} T^2 ) C^2. \end{aligned}$$

Here, while $(n_s,k_s)$ and $(n_d,k_d)$ denote the pairs of refractive index and normalized attenuation coefficient for moist soil and dry soil, $(n_b,k_b)$ and $(n_u,k_u)$ designate the pairs of refractive index and normalized attenuation coefficient for bound soil water and free soil water, respectively; $W_t$ shows the maximum fraction of bound water in soil; *C* indicates the percentage clay content and *T* means the temperature in degrees Celsius.

**Table 3:** Extracted $\varepsilon _{rs}^{\prime }$ by our proposed method (PM), the method in the study^55^, and predicted $\varepsilon _{rs}^{\prime }$ by the Mironov-Fomin model^81,82^ (with percentage difference) at 1.4 GHz for various $\theta _V$ values of all soil samples.

Method	Sample	Parameters								
PM	Soil-A	$\theta _V = 0.17$			$\theta _V = 0.24$			$\theta _V = 0.35$		
		Meas.	Model	$|\Delta \varepsilon _{rs}^{\prime }|$ (%)	Meas.	Model	$|\Delta \varepsilon _{rs}^{\prime }|$ (%)	Meas.	Model	$|\Delta \varepsilon _{rs}^{\prime }|$ (%)
		$9.528 \mp 0.03$	9.763	$2.405 \mp 0.30$	$13.901 \mp 0.03$	14.067	$1.180 \mp 0.21$	$22.771 \mp 0.3$	22.415	$1.588 \mp 0.13$
	Soil-B	$\theta _V = 0.19$			$\theta _V = 0.32$			$\theta _V = 0.44$		
		Meas.	Model	$|\Delta \varepsilon _{rs}^{\prime }|$ (%)	Meas.	Model	$|\Delta \varepsilon _{rs}^{\prime }|$ (%)	Meas.	Model	$|\Delta \varepsilon _{rs}^{\prime }|$ (%)
		$11.170 \mp 0.4$	10.912	$2.364 \mp 0.37$	$20.311 \mp 0.4$	19.946	$1.830 \mp 0.20$	$31.716 \mp 0.5$	30.685	$3.360 \mp 0.17$
^55^	Soil-A	$\theta _V = 0.17$			$\theta _V = 0.24$			$\theta _V = 0.35$		
		Meas.	Model	$|\Delta \varepsilon _{rs}^{\prime }|$ (%)	Meas.	Model	$|\Delta \varepsilon _{rs}^{\prime }|$ (%)	Meas.	Model	$|\Delta \varepsilon _{rs}^{\prime }|$ (%)
		$9.434 \mp 0.05$	9.763	$3.365 \mp 0.50$	$14.334 \mp 0.04$	14.067	$1.898 \mp 0.30$	$22.134 \mp 0.5$	22.415	$1.253 \mp 0.25$
	Soil-B	$\theta _V = 0.19$			$\theta _V = 0.32$			$\theta _V = 0.44$		
		Meas.	Model	$|\Delta \varepsilon _{rs}^{\prime }|$ (%)	Meas.	Model	$|\Delta \varepsilon _{rs}^{\prime }|$ (%)	Meas.	Model	$|\Delta \varepsilon _{rs}^{\prime }|$ (%)
		$10.613 \mp 0.4$	10.912	$2.740 \mp 0.37$	$20.383 \mp 0.4$	19.946	$2.191 \mp 0.20$	$29.472 \mp 0.6$	30.685	$3.953 \mp 0.20$

Table 3 presents a comparison between $\varepsilon _{rs}^{\prime }$ of tested sandy soil samples (with various $\theta _V$ values) extracted by the proposed method and the method in the study^55^ in reference to the data predicted by the Mironov-Fomin model^81,82^. It is seen from the results in Table 3 that extracted $\varepsilon _{rs}^{\prime }$ of tested soil samples by the proposed method and the method in the study^55^ are in good agreement with each other for each $\theta _V$ value. Besides, it is seen from Table 3 that extracted $\varepsilon _{rs}^{\prime }$ of tested sandy soil samples by the proposed method (at maximum 3.4% absolute difference) are closer than those extracted by the method in study^55^ (at maximum 4.0% absolute difference) to the ones predicted by the Mironov-Fomin model^81,82^ and have relatively smaller percentage variations (at maximum 0.37% absolute difference) (obtained from 5 independent measurements) in comparison with those extracted by the method in the study^55^ (at maximum 0.50% absolute difference). We think that this is mainly due to the meniscus formation on top of sandy soil samples while implementing the method in the study^55^, which produces non-flat and uneven sample surface for each independent sample.

After examining the effect of partial meniscus formation on permittivity measurements of sandy soil samples, we continued with the construction of permittivity-moisture curve (calibration curve) using the following linear function^20,27,78^

64 $$\begin{aligned} \theta _V = A \sqrt{\varepsilon _{rs}^{\prime }} - B. \end{aligned}$$

**Figure 10:**
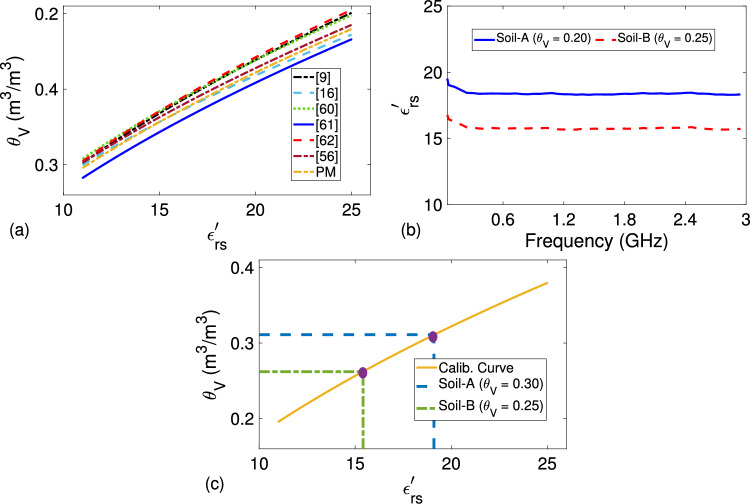
(**a**) Calibration curves constructed by our method (PM) at 3 GHz and the methods in^20,27,55,78–80^, (**b**) frequency dependence of measured $\varepsilon _{rs}^{\prime }$ of Soil-A with $\theta _V = 0.30$ and Soil-B with $\theta _V = 0.25$, and (**c**) mapping $\theta _V$ values from measured $\varepsilon _{rs}^{\prime }$ values using the constructed calibration curve.

Here, *A* and *B* correspond to the constants describing the linear behavior related to measured data (Fig. 7a,b). Utilizing five independent measurements, we computed *A* and *B* fitting parameters aside from $\Delta A$, $\Delta B$, and $R^2$ values, as shown in Table 4 for $f = 3$ GHz. This frequency was selected because S-parameters in hollow coaxial line measurements produce more stable results at higher frequencies^71,78^. For comparison purposes, *A* and *B* values determined by other methods at various frequencies^20,27,55,78–80^ are also given in Table 4. Figure 10a demonstrates calibration curves for our proposed method and all these methods evaluated for $11 \le \varepsilon _{rs}^{\prime } \le 25$ (for demonstration). It is observed from the data in Table 4 and the results in Fig. 10a that the calibration curve constructed by our method is in good agreement with other calibration curves^20,27,55,78–80^ over the entire $11 \le \varepsilon _{rs}^{\prime } \le 25$ range. Our proposed method has relatively smaller $\Delta A$ and $\Delta B$ variations and relatively higher $R^2$ value in comparison with corresponding ones of the methods^27,55^. We think that this is mainly associated with the eliminated effect of meniscus formation on top of tested sandy soil samples by implementing the configurations in Fig. 1a–c and their inverses by our proposed method. For evaluating the effect of inaccurate $L_s$ on the calibration curve, we implemented the method in the study^27^, which necessitates precise information of sample thickness prior to soil permittivity determination, and evaluated the effect of $-5$% variation in $L_s$ on *A*, *B*, $\Delta A$, $\Delta B$, and $R^2$ values. The results are also presented in Table 4. Such an offset introduced a significant change in the calibration curve especially for lower $\theta _V$ or $\varepsilon _{rs}^{\prime }$. For example, determined $\theta _V$ changes approximately from 0.200 to 0.192 (nearly a $4\%$ change) for the minimum $\varepsilon _{rs}^{\prime }$ (e.g., 11.0)^55^.

**Table 4:** Fitted parameters, their standard errors, $R^2$, and RMSE values for $\theta _V$ in (64) using our method (PM) in addition to *A* and *B* values determined by the methods^20,27,55,78–80^.

Method	*A*	$\Delta A$	*B*	$\Delta B$	$R^2$
HP^79^ (50 MHz)	0.109	−	0.1790	−	−
TP^80^ (100MHz)	0.119	−	0.190	−	−
^78^ (390-480 MHz)	0.1130	−	0.1670	−	−
^20^ (3 GHz)	0.1180	0.001	0.1890	0.003	0.9906
^27^ (3 GHz)	0.1025	0.006	0.1403	0.010	0.9872
$L_s$ offset ($-5$%)	0.1065	0.007	0.1611	0.010	0.9866
^55^ (3 GHz)	0.1085	0.005	0.1574	0.007	0.9894
PM (3 GHz)	0.1092	0.004	0.1664	0.005	0.9901

Here, HP and TP refer to Hydra Probe and Theta Probe calibrations, respectively

Finally, we assessed the performance of the calibration curve constructed by our method for predicting $\theta _V$ value from measured $\varepsilon _{rs}^{\prime }$. Figure 10b illustrates extracted $\varepsilon _{rs}^{\prime }$ values of Soil-A with $\theta _V = 0.30$ ($\varepsilon _{rs}^{\prime } \cong 19.097$) and Soil-B with $\theta _V = 0.25$ ($\varepsilon _{rs}^{\prime } \cong 15.407$) by our proposed method. When extracted values at 3 GHz in Fig. 10b were utilized in (64), predicted $\theta _V$ values, as shown in Fig. 10c, were approximately found to be 0.311 and 0.262, respectively, resulting a percentage difference of $|\Delta \theta _V| = 3.601$% and $|\Delta \theta _V| = 4.891$% in reference to known $\theta _V$ known a priori. It is seen from these results that our calibration curve predicts $\theta _V$ values within $5\%$ margin (Table 6).

**Table 5:** Comparison of our proposed method with some calibration-dependent and calibration-independent non-resonant microwave methods in the literature.

Method	Calib.	Sample type	Thickness information	Meniscus problem	Theoretical model	Number of measurement configurations (How They Are Implemented)
^39,41^	**Yes**	**Solid Only**	**Needed**	−	S-Parameters	One (Sample Only)
^42^	**Yes**	**Solid Only**	**Needed**	−	S-Parameters	Sample and Empty Line
^51^	**Yes**	Granular or Liquid	**Needed**	**Yes**	S-Parameters	One (Sample on One Holder)
^52,53^	**Yes**	Granular or Liquid	**Needed**	**Yes**	S-Parameters	Two (Holder Only and Sample on One Holder)
^54^	**Yes**	Granular or Liquid	**Needed**	**Yes**	S-Parameters	Three (Holder Only and Two Identical Samples with Different Lengths (Separate) on One Holder)
^19,20^	**Yes**	Granular or Liquid	**Needed**	No	WCM or T-Matrix	Two (Sample (or Air) Sandwiched Between Two Holders)
^21^	**Yes**	Granular or Liquid	**Needed**	No	S-Parameters	One (Sample Sandwiched Between Two Holders)
^22,23^	**Yes**	Granular or Liquid	**Needed**	No	S-Parameters	One (Sample Sandwiched Between Two Holders)
^24^	**Yes**	Granular or Liquid	**Needed**	No	S-Parameters (High-Freq.) and Lump Parameters (Low-Freq.)	One (Sample Sandwiched Between Two Holders)
^25^	**Yes**	Granular or Liquid	**Needed**	No	S-Parameters	Electronic Control Unit (Multiple) (Sample Sandwiched Between Two Holders)
^40^	**Yes**	Granular or Liquid	**Needed**	No	S-Parameters Along With ABCD Matrix	One (Sample Sandwiched Between Two Holders)
^43^	**Yes**	Granular or Liquid	**Needed**	No	S-Parameters	One (Sample Sandwiched Between Two Holders)
^44,45^	**Yes**	Granular or Liquid	**Needed**	No	S-Parameters	One (Sample Sandwiched Between Thin Films)
^46^	**Yes**	Granular or Liquid	**Needed**	No	S-Parameters Along With ABCD Matrix	One (Sample Sandwiched Between Two Holders)
^47,48^	**Yes**	Granular or Liquid	**Needed**	No	S-Parameters	One (Sample Sandwiched Between Two Holders)
^49^	**Yes**	Granular or Liquid	No	No	S-Parameters	One (Sample Sandwiched Between Two Holders)
^50^	**Yes**	Granular or Liquid	No	No	S-Parameters	Three (Holder Only and Two Identical Samples with Different Lengths (Separate) on One Holder)
^56^	No	**Solid Only**	**Needed**	−	WCM	Two (Two Identical Samples With Different Lengths)
^57^	No	**Solid Only**	**Needed**	−	WCM	Five (Two Different Samples With Different Lengths (Direct and Reversed Config.) and Empty Line)
^60^	No	**Solid Only**	**Needed**	−	WCM	Two (One Sample Inside Lines With Different Lengths)
^61^	No	**Solid Only**	**Needed**	−	WCM	Two (Sample-Only and Empty Line)

**Table 6:** Comparison of our proposed method with some calibration-dependent and calibration-independent non-resonant microwave methods in the literature.

Method	Calib.	Sample type	Thickness information	Meniscus problem	Theoretical model	Number of Measurement Configurations (How They Are Implemented)
^62^	No	**Solid Only**	**Needed**	−	WCM	Two (Two Identical Samples With Different Lengths)
^65^	No	**Solid Only**	**Needed**	−	WCM	Three (Thru Connection, Empty Line, and One Sample Inside the Line)
^64^	No	**Solid Only**	No	−	WCM	Three (Empty Line and Direct and Reversed Line Loaded With One Sample)
^58^	No	Granular or Liquid	**Needed**	**Yes**	WCM	Two (Two Identical Samples With Different Lengths)
^69^	No	Granular or Liquid	**Needed**	**Yes**	WCM	Two (Holder Only and Sample on the Holder)
^59^	No	Granular or Liquid	**Needed**	**Yes**	WCM	Two (Thru Connection and Sample Sandwiched Between Two Holders)
^70^	No	Granular or Liquid	**Needed**	**Yes**	WCM	Three (First Holder Only, Sample on This Holder, and Sample Sandwiched Between Two Holders)
^66^	No	Granular or Liquid	**Needed**	**Yes**	WCM	Three (Thru Connection, Empty Line, and Sample on One Holder in this Line)
^63^	No	Granular or Liquid	**Needed**	No	WCM	Two (Empty Line With Two Identical Sample Holders and Sample Sandwiched Between These Holders)
^67^	No	Granular or Liquid	**Needed**	No	TM (or ABCD)	Two (Empty Line With Two Identical Sample Holders and Sample Sandwiched Between These Holders)
^68^	No	Granular or Liquid	**Needed**	No	TM (or ABCD)	Two (Empty Line and Sample Sandwiched Between Non-Identical Sample Holders)
^71^	No	Granular or Liquid	**Needed**	No	WCM	Three (Three Identical Samples With Different Lengths on the Same Sample Holder)
^55^	No	Granular or Liquid	No	**Yes**	WCM	Four (Empty Line, Direct and Reversed Configurations of Holder Loaded Line, and Sample on this Holder in the Line)
Proposed	No	Granular or Liquid	No	No	WCM and STM	Three (Empty Line, Sample Sandwiched Between Non-Identical Sample Holders in the Line, and its Shifted Configuration in the Line)

The study in^58^ is also applicable for granular or liquid samples, but requiring thickness information and having meniscus problem.

## Advantages and limitations of our method and its comparison

Table 5 illustrates a comparison of some calibration-dependent and calibration-independent non-resonant microwave methods in the literature in terms of the need for calibration, sample type, whether the extraction method needs information about the sample thickness, whether the meniscus problem is being solved for granular/liquid samples, the model the theoretical analysis is performed, the number of measurement configurations, and how they are implemented. It is seen from Table 5 that the methods in the studies^19–25,39–54^ are dependent on the perfection of calibration standards used in the calibration process. Therefore, their accuracy relies heavily on a formal calibration procedure. On the other hand, the methods in the studies^55–71^ do not require a formal calibration procedure (calibration-independent), thereby eliminating the effect of imperfect calibration standards on measurements. When these calibration-independent are examined, it is noted that the methods in the studies^56,57,60–62,64,65^ are applicable for material property analysis of solid materials,those in the studies^55,58,59,63,66–71^ are feasible for material characterization of granular/liquid samples. Besides,the methods in the studies^19–25,39–48,51–54,56–63,65–71^ require information of sample thickness to determine its electromagnetic parameter. It is worthy to note that such a requirement can influence the calibration-curve parameters,resulting in inaccurate soil moisture determination. Additionally,while the methods in the studies^19–25,40,43–50,63,67,68,71^ suffer from meniscus formation on top of liquid samples or samples composed of a mixture of granular form and liquid state,the methodologies in the studies^19–25,40,43–50,63,67,68,71^ have the potential of eliminating,if not reducing,the effect of this meniscus problem by means of utilizing two plugs sandwiching the sample or through performing relative measurements of liquid samples with different lengths having identical meniscus formation. As different from the methods in previous studies^19–25,39–71^, our proposed method comes the forefront in consideration of the unique features that it eliminates the need for a formal calibration procedure, is applicable for electromagnetic characterization of liquid samples or samples involving a mixture of granular form and liquid state, does not require thickness information for such a characterization, and eliminates the effect of meniscus formation present over top of most granular/liquid samples on their electromagnetic characterization. Besides, its theoretical model unifies the WCM and STM analyses, distinguishing it from the analyses performed in the studies^19–25,39–71^ which either use S-Parameters only, WCM only, or TM only. Furthermore, even though it requires three different measurement configurations, it necessitates one sample and one holder, differing from other methods^50,54,56–58,62,63,67,71^ which use at least two samples. It should be mentioned that using more than one sample for this electromagnetic characterization introduces additional measurement uncertainty due to sample length uncertainty and inhomogeneity. Nonetheless, our method requires a special care in preparing homogeneous sandy soil samples over the length of the coaxial line. Besides, attention is needed before positioning the top plug on the poured sandy soil samples into the measurement coaxial cell to have a bubble-free sample by pouring samples slowly and uniformly. Furthermore, as noted from the dependencies in Fig. 9a,b, precise information of $L_{01}$ should be provided for our extraction method to produce accurate $\varepsilon _{rs}$. On the other hand, although special attention was paid on preparing a homogeneous sandy soil and water composite mixture to attain different moisture levels by means of a slow-mixing and pouring each prepared mixture into the hollow coaxial line via a small tilt procedure, some air bubbles may appear in a prepared mixture if this attention was not implemented. For such cases, there might be air bubbles not only in the transverse plane but also in the wave propagation direction so that their effects need to be taken into account. For more information, the methodologies in the following recent studies^83–85^ can be exercised if needed. Finally, the proposed method was tested and thereby is applicable for $\varepsilon _{rs}$ measurements of sandy soil samples. Furthermore, it requires the soil samples to be tested to have considerable moisture level so that the plug-soil-plug combination in Fig. 1b could be easy movable by some distance $L_{01}$ to arrive at the plug-soil-plug combination in Fig. 1c. Any extraction method operating at microwave frequencies and feasible for soil samples with relatively lower moisture level and with different textures including silty soil, clay soil, loam soil, or organic soil would be attractive, which is a subject of our future research plan.

## Conclusion

A solid theoretical model combining the WCM and STM presentations is harnessed to determine permittivity of granular/liquid samples (e.g., sandy soil samples) from microwave-driven hollow coaxial line measurements. The extraction method unifies into one extraction model the following features of calibration-independence, thickness-independence, and meniscus-free characterization. The extraction method uses relatively simple configurations of empty line, a (sandy) soil sample sandwiched between two identical plugs, and shifted version of this plug-sample-plug structure, including their inverse configurations through port switching operation. After validating the meniscus-free and thickness-independence features of the proposed extraction method, which reduces absolute value difference of $\varepsilon _{rs}^{\prime }$, by means of two reference liquid samples (distilled water and methanol), a soil-permittivity curve, supplemented by the three-pole Debye and the Mironov-Fomin models, is established through permittivity measurements of two different sandy soil samples with various $\theta _V$ values. This constructed curve is tested by predicting $\theta _V$ of the tested sandy soil samples by way of $\varepsilon _{rs}^{\prime }$ measurement, producing results within $5\%$ margin of $\theta _V$. Our proposed method can find diverse applications in calibration-free characterization of granular/liquid samples wherever thickness-information is a necessity and meniscus formation is critical.

## Data Availability

The datasets used and analyzed during the current study are available from the corresponding author (U.C.H.) upon reasonable request

## References

[1] Lekshmi, S. S., Singh, D. & Baghini, M. A critical review of soil moisture measurement. *Measurement* **54**, 92–105 (2014).

[2] Ochsner, T. E. et al. State of the art in large-scale soil moisture monitoring. *Soil Sci. Soc. Am. J.* **77**, 1888–1919. 10.2136/sssaj2013.03.0093 (2013).

[3] Hupet, F. & Vanclooster, M. Intraseasonal dynamics of soil moisture variability within a small agricultural maize cropped field. *J. Hydrol.* **261**, 86–101. 10.1016/S0022-1694(02)00016-1 (2002).

[4] Paul, W. Prospects for controlled application of water and fertiliser, based on sensing permittivity of soil. *Comput. Electron. Agric.* **36**, 151–163. 10.1016/S0168-1699(02)00098-4 (2002).

[5] Sheng, W. et al. Considerations on measurement frequency of electromagnetic sensors for soil water content determination. *Geoderma* **457**, 117292. 10.1016/j.geoderma.2025.117292 (2025).

[6] Robinson, D. A. et al. Soil moisture measurement for ecological and hydrological watershed-scale observatories: A review. *Vadose Zone J.* **7**, 358–389, 10.2136/vzj2007.0143 (2008).

[7] S.U., S. L., Singh, D. & Shojaei Baghini, M. A critical review of soil moisture measurement. *Measurement* **54**, 92–105. 10.1016/j.measurement.2014.04.007 (2014).

[8] Yu, L. et al. Review of research progress on soil moisture sensor technology. *Int. J. Agric. Biol. Eng.* **14**, 32–42 (2021).

[9] Hasar, H. *Assessment of moisture contents of sand textured soils using microwave calibration-independent methods,” Ph.D dissertation*. Dissertation, Natural Science Institute, Ataturk University (2022).

[10] Hasar, H. On various soil moisture measurement techniques available in the literature. *Black Sea J. Agric.* **7**, 570–579 (2024).

[11] Singh, D. N. & Kuriyan, S. J. Estimation of unsaturated hydraulic conductivity using soil suction measurements obtained by an insertion tensiometer. *Can. Geotech. J.* **40**, 476–483 (2003).

[12] Topp, G. C., Davis, J. L. & Annan, A. P. Electromagnetic determination of soil water content: Measurements in coaxial transmission lines. *Water Resour. Res.* **16**, 574–582. 10.1029/WR016i003p00574 (1980).

[13] Majcher, J. et al. Application of a dagger probe for soil dielectric permittivity measurement by tdr. *Measurement* **178**, 109368. 10.1016/j.measurement.2021.109368 (2021).

[14] Cristi, F., Fierro, V., Suárez, F., Muñoz, J. F. & Hausner, M. B. A tdr-waveform approach to estimate soil water content in electrically conductive soils. *Comput. Electron. Agric.* **121**, 160–168. 10.1016/j.compag.2015.12.004 (2016).

[15] Mendoza Veirana, G., Verhegge, J., Cornelis, W. & De Smedt, P. Soil dielectric permittivity modelling for 50 mhz instrumentation. *Geoderma* **438**, 116624. 10.1016/j.geoderma.2023.116624 (2023).

[16] Babaeian, E. et al. Ground, proximal, and satellite remote sensing of soil moisture. *Rev. Geophys.* **57**, 530–616. 10.1029/2018RG000618 (2019).

[17] Wagner, N., Schwing, M. & Scheuermann, A. Numerical 3-d fem and experimental analysis of the open-ended coaxial line technique for microwave dielectric spectroscopy on soil. *IEEE Trans. Geosci. Remote Sens.* **52**, 880–893. 10.1109/TGRS.2013.2245138 (2014).

[18] Bore, T. et al. Coaxial method to investigate broadband dielectric properties of rocks over the 5 hz to 3 ghz frequency range. *J. Rock Mech. Geotech. Eng.*10.1016/j.jrmge.2025.04.024 (2025).

[19] Lewandowski, A. et al. 0.05–3 ghz vna characterization of soil dielectric properties based on the multiline trl calibration. *Meas. Sci. Technol.* **28**, 024007, 10.1088/1361-6501/28/2/024007 (2017).

[20] Szyplowska, A. et al. Impact of soil salinity, texture and measurement frequency on the relations between soil moisture and 20 mhz-3 ghz dielectric permittivity spectrum for soils of medium texture. *J. Hydrol.* **579**, 124155 (2019).

[21] Ba, D. & Sabouroux, P. Epsimu, a toolkit for permittivity and permeability measurement in microwave domain at real time of all materials: Applications to solid and semisolid materials. *Microw. Opt. Technol. Lett.* **52**, 2643–2648. 10.1002/mop.25570 (2010).

[22] Wagner, N., Emmerich, K., Bonitz, F. & Kupfer, K. Experimental investigations on the frequency- and temperature-dependent dielectric material properties of soil. *IEEE Trans. Geosci. Remote Sens.* **49**, 2518–2530. 10.1109/TGRS.2011.2108303 (2011).

[23] Lauer, K., Wagner, N. & Felix-Henningsen, P. A new technique for measuring broadband dielectric spectra of undisturbed soil samples. *Eur. J. Soil Sci.* **63**, 224–238. 10.1111/j.1365-2389.2012.01431.x (2012).

[24] Bobrov, P. P., Repin, A. V. & Rodionova, O. V. Wideband frequency domain method of soil dielectric property measurements. *IEEE Trans. Geosci. Remote Sens.* **53**, 2366–2372. 10.1109/TGRS.2014.2359092 (2015).

[25] Lewandowski, A. et al. One-port vector network analyzer characterization of soil dielectric spectrum. *IEEE Trans. Geosci. Remote Sens.* **57**, 3661–3676. 10.1109/TGRS.2018.2886474 (2019).

[26] Hasar, H. et al. Broadband soil permittivity measurements using a novel de-embedding line-line method. *IEEE Geosci. Remote Sens. Lett.* **19**, 1–5. 10.1109/LGRS.2021.3140097 (2022).

[27] Hasar, H. et al. Permittivity extraction of soil samples using coaxial-line measurements by a simple calibration. *IEEE Trans. Geosci. Remote Sens.* **61**, 1–8. 10.1109/TGRS.2022.3233912 (2023).

[28] Keshavarz, R. & Shariati, N. High-sensitivity and compact time domain soil moisture sensor using dispersive phase shifter for complex permittivity measurement. *IEEE Trans. Instrum. Meas.* **71**, 1–10. 10.1109/TIM.2021.3132367 (2022).

[29] Noborio, K. Measurement of soil water content and electrical conductivity by time domain reflectometry: a review. *Comput. Electron. Agric.* **31**, 213–237. 10.1016/S0168-1699(00)00184-8 (2001).

[30] Maleki Gargari, A., Zarifi, M. H. & Markley, L. Passive matched mushroom structure for a high sensitivity low profile antenna-based material detection system. *IEEE Sensors J.* **19**, 6154–6162. 10.1109/JSEN.2019.2908687 (2019).

[31] Frau, I. et al. Microwave sensors for in situ monitoring of trace metals in polluted water. *Sensors* **21**, 10.3390/s21093147 (2021). 10.3390/s21093147 PMC8125159 34062849

[32] Frau, I. et al. Detection of zn in water using novel functionalised planar microwave sensors. *Mater. Sci. Eng., B* **247**, 114382. 10.1016/j.mseb.2019.114382 (2019).

[33] Krivosudský, O., Havelka, D., Chafai, D. E. & Cifra, M. Microfluidic on-chip microwave sensing of the self-assembly state of tubulin. *Sens. Actuators, B Chem.* **328**, 129068. 10.1016/j.snb.2020.129068 (2021).

[34] Mane, S., Das, N., Singh, G., Cosh, M. & Dong, Y. Advancements in dielectric soil moisture sensor calibration: A comprehensive review of methods and techniques. *Comput. Electron. Agric.* **218**, 108686. 10.1016/j.compag.2024.108686 (2024).

[35] Szypłowska, A. et al. Dielectric models for moisture determination of soils with variable organic matter content. *Geoderma* **401**, 115288. 10.1016/j.geoderma.2021.115288 (2021).

[36] Kaatze, U. Techniques for measuring the microwave dielectric properties of materials. *Metrologia* **47**, S91. 10.1088/0026-1394/47/2/S10 (2010).

[37] Woszczyk, A. et al. An open-ended probe with an antenna for the measurement of the water content in the soil. *Comput. Electron. Agric.* **167**, 105042. 10.1016/j.compag.2019.105042 (2019).

[38] González-Teruel, J. D. et al. Measurement of the broadband complex permittivity of soils in the frequency domain with a low-cost vector network analyzer and an open-ended coaxial probe. *Comput. Electron. Agric.* **195**, 106847. 10.1016/j.compag.2022.106847 (2022).

[39] Ghodgaonkar, D., Varadan, V. & Varadan, V. Free-space measurement of complex permittivity and complex permeability of magnetic materials at microwave frequencies. *IEEE Trans. Instrum. Meas.* **39**, 387–394. 10.1109/19.52520 (1990).

[40] Jose, K. A., Varadan, V. K. & Varadan, V. V. Wideband and noncontact characterization of the complex permittivity of liquids. *Microw. Opt. Technol. Lett.* **30**, 75–79. 10.1002/mop.1225 (2001).

[41] Nicolson, A. M. & Ross, G. F. Measurement of the intrinsic properties of materials by time-domain techniques. *IEEE Trans. Instrum. Meas.* **19**, 377–382. 10.1109/TIM.1970.4313932 (1970).

[42] Chalapat, K., Sarvala, K., Li, J. & Paraoanu, G. S. Wideband reference-plane invariant method for measuring electromagnetic parameters of materials. *IEEE Trans. Microw. Theory Tech.* **57**, 2257–2267. 10.1109/TMTT.2009.2027160 (2009).

[43] Bois, K., Handjojo, L., Benally, A., Mubarak, K. & Zoughi, R. Dielectric plug-loaded two-port transmission line measurement technique for dielectric property characterization of granular and liquid materials. *IEEE Trans. Instrum. Meas.* **48**, 1141–1148. 10.1109/19.816128 (1999).

[44] Wang, Y. & Afsar, M. N. Measurement of complex permittivity of liquid dielectrics. *Microw. Opt. Technol. Lett.* **34**, 240–243. 10.1002/mop.10427 (2002).

[45] Wang, Y. & and, M. N. A. Measurement of complex permittivity of liquids using waveguide techniques - abstract. *J. Electromagn. Waves Appl.* **17**, 1311–1312. 10.1163/156939303322520052 (2003).

[46] Ogunlade, O., Pollard, R. & Hunter, I. A new method of obtaining the permittivity of liquids using in-waveguide technique. *IEEE Microwave Wirel. Compon. Lett.* **16**, 363–365. 10.1109/LMWC.2006.875594 (2006).

[47] Hasar, U. C. A fast and accurate amplitude-only transmission-reflection method for complex permittivity determination of lossy materials. *IEEE Trans. Microw. Theory Tech.* **56**, 2129–2135. 10.1109/TMTT.2008.2002229 (2008).

[48] Hasar, U. C., Westgate, C. R. & Ertugrul, M. Noniterative permittivity extraction of lossy liquid materials from reflection asymmetric amplitude-only microwave measurements. *IEEE Microwave Wirel. Compon. Lett.* **19**, 419–421. 10.1109/LMWC.2009.2020045 (2009).

[49] Hasar, U. C. & Cansiz, A. Simultaneous complex permittivity and thickness evaluation of liquid materials from scattering parameter measurements. *Microw. Opt. Technol. Lett.* **52**, 75–78. 10.1002/mop.24837 (2010).

[50] Kalisiak, M. & Wiatr, W. Complete meniscus removal method for broadband liquid characterization in a semi-open coaxial test cell. *Sensors* **19**. 10.3390/s19092092 (2019). 10.3390/s19092092 PMC6539370 31064077

[51] Hasar, U. C., Kaya, Y., Bute, M., Barroso, J. J. & Ertugrul, M. Microwave method for reference-plane-invariant and thickness-independent permittivity determination of liquid materials. *Rev. Sci. Instrum.* **85**, 014705. 10.1063/1.4862047 (2014). 24517796 10.1063/1.4862047

[52] Hasar, U. C., Kaya, Y., Barroso, J. J. & Ertugrul, M. Determination of reference-plane invariant, thickness-independent, and broadband constitutive parameters of thin materials. *IEEE Trans. Microw. Theory Tech.* **63**, 2313–2321. 10.1109/TMTT.2015.2431685 (2015).

[53] Hasar, U. & Bute, M. Reference-invariant permittivity and thickness measurement of lossy liquid materials. *Electron. Lett.* **53**, 544–546. 10.1049/el.2016.3793 (2017).

[54] Kalisiak, M. & Wiatr, W. Errors in broadband permittivity determination due to liquid surface distortions in semi-open test cell. *Remote Sens.* **13**. 10.3390/rs13050983 (2021).

[55] Hasar, H., Hasar, U. C., Korkmaz, H., Balasubramanian, V. & Zarifi, M. H. Extracting soil permittivity using uncalibrated, thickness and position-independent hollow coaxial-probe techniques. *IEEE Trans. Microw. Theory Tech.* **73**, 3930–3938. 10.1109/TMTT.2024.3523239 (2025).

[56] Janezic, M. & Jargon, J. Complex permittivity determination from propagation constant measurements. *IEEE Microwave Guided Wave Lett.* **9**, 76–78. 10.1109/75.755052 (1999).

[57] Wan, C., Nauwelaers, B., De Raedt, W. & Van Rossum, M. Two new measurement methods for explicit determination of complex permittivity. *IEEE Trans. Microw. Theory Tech.* **46**, 1614–1619. 10.1109/22.734537 (1998).

[58] Huynen, I., Steukers, C. & Duhamel, F. A wideband line-line dielectrometric method for liquids, soils, and planar substrates. *IEEE Trans. Instrum. Meas.* **50**, 1343–1348. 10.1109/19.963208 (2001).

[59] Hasar, U. C. A calibration-independent method for accurate complex permittivity determination of liquid materials. *Rev. Sci. Instrum.* **79**, 086114. 10.1063/1.2976037 (2008). 19044395 10.1063/1.2976037

[60] Hasar, U. C. A new calibration-independent method for complex permittivity extraction of solid dielectric materials. *IEEE Microwave Wirel. Compon. Lett.* **18**, 788–790. 10.1109/LMWC.2008.2007699 (2008).

[61] Caijun, Z., Quanxing, J. & Shenhui, J. Calibration-independent and position-insensitive transmission/reflection method for permittivity measurement with one sample in coaxial line. *IEEE Trans. Electromagn. Compat.* **53**, 684–689. 10.1109/TEMC.2011.2156416 (2011).

[62] Jebbor, N., Bri, S., Sánchez, A. & Chaibi, M. A fast calibration-independent method for complex permittivity determination at microwave frequencies. *Measurement* **46**, 2206–2209. 10.1016/j.measurement.2013.04.009 (2013).

[63] Guoxin, C. Calibration-independent measurement of complex permittivity of liquids using a coaxial transmission line. *Rev. Sci. Instrum.* **86**, 014704. 10.1063/1.4905362 (2015). 25638105 10.1063/1.4905362

[64] Hasar, U. C. Thickness-invariant complex permittivity retrieval from calibration-independent measurements. *IEEE Microwave Wirel. Compon. Lett.* **27**, 201–203. 10.1109/LMWC.2016.2647000 (2017).

[65] Hasar, U. C. Self-calibrating transmission-reflection technique for constitutive parameters retrieval of materials. *IEEE Trans. Microw. Theory Tech.* **66**, 1081–1089. 10.1109/TMTT.2017.2756964 (2018).

[66] Hasar, U. C. & Kaya, Y. Self-calibrating noniterative complex permittivity extraction of thin dielectric samples. *IEEE Trans. Electromagn. Compat.* **60**, 354–361. 10.1109/TEMC.2017.2715161 (2018).

[67] Hasar, H. et al. Prediction of water-adulteration within honey by air-line de-embedding waveguide measurements. *Measurement* **179**, 109469. 10.1016/j.measurement.2021.109469 (2021).

[68] Hasar, H. et al. Honey–water content analysis by mixing models using a self-calibrating microwave method. *IEEE Trans. Microw. Theory Tech.* **71**, 691–697. 10.1109/TMTT.2022.3208024 (2023).

[69] Hasar, U. Calibration-independent method for complex permittivity determination of liquid and granular materials. *Electron. Lett.* **44**, 585–587. 10.1049/el:20080242 (2008). 10.1063/1.2976037 19044395

[70] Hasar, U., Simsek, O., Zateroglu, M. & Ekinci, A. A microwave method for unique and non-ambiguous permittivity determination of liquid materials from measured uncalibrated scattering parameters. *Prog. Electromagn. Res.* **95**, 73–85 (2009).

[71] Kalisiak, M., Lewandowski, A. & Wiatr, W. Meniscus-corrected method for broadband liquid permittivity measurements with an uncalibrated vector network analyzer. *Sensors* **23**, 10.3390/s23125401 (2023). 10.3390/s23125401 PMC10302224 37420567

[72] Zarifi, D., Soleimani, M. & Abdolali, A. Electromagnetic characterization of uniaxial chiral composites using state transition matrix method. *IEEE Trans. Antennas Propag.* **61**, 5658–5665. 10.1109/TAP.2013.2277858 (2013).

[73] Hasar, U. C., Ozturk, G., Kaya, Y., Barroso, J. J. & Ertugrul, M. Simple and accurate electromagnetic characterization of omega-class bianisotropic metamaterials using the state transition matrix method. *IEEE Trans. Antennas Propag.* **69**, 7064–7067. 10.1109/TAP.2021.3098550 (2021).

[74] Mosavirik, T., Hashemi, M., Soleimani, M., Nayyeri, V. & Ramahi, O. M. Accuracy-improved and low-cost material characterization using power measurement and artificial neural network. *IEEE Trans. Instrum. Meas.* **70**, 1–9. 10.1109/TIM.2021.3126011 (2021).33776080

[75] González-Teruel, J. D. et al. Dielectric spectroscopy and application of mixing models describing dielectric dispersion in clay minerals and clayey soils. *Sensors* **20**. 10.3390/s20226678 (2020). 10.3390/s20226678 PMC7700415 33266418

[76] Vasilyeva, M. et al. Dielectric relaxation of water in clay minerals. *Clays Clay Miner.* **62**, 62–73. 10.1346/CCMN.2014.0620106 (2014).

[77] Chen, Y. & Or, D. Geometrical factors and interfacial processes affecting complex dielectric permittivity of partially saturated porous media. *Water Resour. Res.* **42**. 10.1029/2005WR004744 (2006).

[78] Skierucha, W. & Wilczek, A. A fdr sensor for measuring complex soil dielectric permittivity in the 10–500 mhz frequency range. *Sensors* **10**, 3314–3329. 10.3390/s100403314 (2010). 22319300 10.3390/s100403314 PMC3274183

[79] Vaz, C. M., Jones, S., Meding, M. & Tuller, M. Evaluation of standard calibration functions for eight electromagnetic soil moisture sensors. *Vadose Zone J.* **12**, vzj2012.0160. 10.2136/vzj2012.0160 (2013).

[80] Devices, D.-T. User manual for the ml3 thetaprobe. *Delta-T Devices Ltd.* (2017).

[81] Mironov, V. & Fomin, S. Temperature and mineralogy dependable model for microwave dielectric spectra of moist soils. *Proc. PIERS* 938–942 (2009).

[82] Valery, M., Yann, K., Jean-Pierre, W., Liudmila, K. & François, D. Temperature and texture dependent dielectric model of moist soils at the smos frequency. In *2012 IEEE International Geoscience and Remote Sensing Symposium*, 1127–1130, 10.1109/IGARSS.2012.6351350 (2012).

[83] Computation of electric and magnetic field distribution inside a multilayer cylindrical conductor. *Prog. Electromagn. Res. M* **88**, 53–63, 10.2528/PIERM19101702 (2020).

[84] Kubiczek, K. Computation of the characteristic parameters of coaxial waveguides used in precision sensors. *Sensors* **23**, 10.3390/s23042324 (2023). 10.3390/s23042324 PMC9961928 36850922

[85] Wang, Y., Hooper, I., Edwards, E. & Grant, P. S. Gap-corrected thin-film permittivity and permeability measurement with a broadband coaxial line technique. *IEEE Trans. Microw. Theory Tech.* **64**, 924–930. 10.1109/TMTT.2016.2519915 (2016).

